# Deregulated expression of NKL homeobox genes in T-cell lymphomas

**DOI:** 10.18632/oncotarget.26929

**Published:** 2019-05-14

**Authors:** Stefan Nagel, Claudia Pommerenke, Roderick A.F. MacLeod, Corinna Meyer, Maren Kaufmann, Silke Fähnrich, Hans G. Drexler

**Affiliations:** ^1^Department of Human and Animal Cell Lines, Leibniz-Institute DSMZ–German Collection of Microorganisms and Cell Cultures, Braunschweig, Germany

**Keywords:** homeobox, HSTL, NKL-code, T-ALL

## Abstract

Recently, we have presented a scheme, termed “NKL-code”, which describes physiological expression patterns of NKL homeobox genes in early hematopoiesis and in lymphopoiesis including main stages of T-, B- and NK-cell development. Aberrant activity of these genes underlies the generation of hematological malignancies notably T-cell leukemia. Here, we searched for deregulated NKL homeobox genes in main entities of T-cell lymphomas comprising angioimmunoblastic T-cell lymphoma (AITL), anaplastic large cell lymphoma (ALCL), adult T-cell leukemia/lymphoma (ATLL), hepatosplenic T-cell lymphoma (HSTL), NK/T-cell lymphoma (NKTL) and peripheral T-cell lymphoma (PTCL). Our data revealed altogether 19 aberrantly overexpressed genes in these types, demonstrating deregulated NKL homeobox genes involvement in T-cell lymphomas as well. For detailed analysis we focused on NKL homeobox gene MSX1 which is normally expressed in NK-cells. MSX1 was overexpressed in subsets of HSTL patients and HSTL-derived sister cell lines DERL-2 and DERL-7 which served as models to characterize mechanisms of deregulation. We performed karyotyping, genomic and expression profiling, and whole genome sequencing to reveal mutated and deregulated gene candidates, including the fusion gene CD53-PDGFRB. Subsequent knockdown experiments allowed the reconstruction of an aberrant network involved in MSX1 deregulation, including chromatin factors AUTS2 and mutated histone HIST1H3B(K27M). The gene encoding AUTS2 is located at chromosome 7q11 and may represent a basic target of the HSTL hallmark aberration i(7q). Taken together, our findings highlight an oncogenic role for deregulated NKL homeobox genes in T-cell lymphoma and identify MSX1 as a novel player in HSTL, implicated in aberrant NK- and T-cell differentiation.

## INTRODUCTION

All types of lymphocytes are generated in the course of hematopoiesis which starts with hematopoietic stem cells (HSC) and the derived common lymphoid progenitors (CLP) in the bone marrow. The subsequent differentiation of B-cells and NK-cells persists in this compartment while early T-cell progenitors (ETP) move into the thymus to complete their differentiation. The developmental steps in early hematopoiesis and in lymphopoiesis are primarily regulated at the transcriptional level [1]. Accordingly, transcription factors (TFs) like TCF3/E2A and TAL1 control early hemic processes while BCL11B, PAX5 and ID2 subsequently regulate the respective differentiation of T-cells, B-cells and NK-cells [1–3].

Homeobox genes encode TFs which are involved in basic operations of cell, tissue, and organ differentiation in the embryo and in the adult. NKL homeobox genes represent a subclass of this group and comprise 48 members, including NKX2-3 and NKX2-5 which operate as master genes in the developing spleen and heart, respectively [4–6]. Recently, we proposed the term “NKL-code” to describe the physiological roles of all NKL homeobox genes normally expressed in early hematopoiesis and in lymphopoiesis [7, 8]. To this code belong HHEX, HLX, NKX2-3 and NKX3-1 which are active in HSCs, HHEX, HLX and MSX1 in CLPs, and MSX1 in mature NK-cells. HHEX, HLX, NKX3-1, TLX2 and VENTX are expressed in early stages of T-cell development, contrasting with late stages and mature T-cells which lack NKL homeobox gene activity. This circumstance may underlie the malignant susceptibility of T-cells to aberrantly express NKL homeobox genes and undergo developmental arrest at immature stages [7, 9]. Accordingly, 24 aberrantly activated genes of this subclass have been described in T-cell leukemia patients to date. These oncogenes include both NKL-code members and ectopically expressed non-code members normally silent in hematopoiesis [7, 10].

Mechanisms of NKL homeobox gene deregulation include recurrent chromosomal rearrangements juxtaposing NKL genes with those encoding the T-cell receptors (TCR) or BCL11B, aberrant activities of signalling pathways including the BMP-pathway, failed downregulation of upstream factors normally operating in early stages of hematopoiesis only, and aberrant alterations of chromatin components including histones and AUTS2 [11–15]. Chromatin-associated polycomb repressor complexes (PRC) are important regulators of homeobox genes in development. EZH2 constitutes the enzymatic component of PRC2 and performs tri-methylation of histone H3 at position K27 which results in gene silencing [16]. PCGF5 belongs to the complex subtype PRC1.5 which mediates gene silencing via ubiquitinylation of histone H2A [17]. AUTS2 interacts with PCGF5 and transforms the suppressive complex into an activator which recruits histone acetyltransferase [18]. This function contributes to both, normal activity of NKL homeobox gene MSX1 in NK-cells and aberrant expression in T-cell acute lymphoid leukemia (T-ALL) [15, 19].

Here, we extended our analysis of aberrantly activated NKL homeobox genes to mature T-cell and NK-cell neoplasms. According to the WHO classification, this group of malignancies comprises several entities including the most common type peripheral T-cell lymphoma (PTCL) and the rare type hepatosplenic T-cell lymphoma (HSTL) [20, 21]. Many T-cell lymphomas carry poor outcomes demanding identification of novel therapeutic targets and options which require determination of the molecular drivers of these neoplasms. We screened public patient datasets for deregulated NKL homeobox genes and focused experimentally on MSX1 using two HSTL-derived sister cell lines as a model. This study documents an oncogenic role for these genes in T-cell lymphomas and reveals aberrant regulatory gene networks upstream of MSX1 and gene expression changes attributable to tumor evolution.

## RESULTS

### Aberrant NKL homeobox gene activities in T-cell lymphomas

NKL homeobox genes are silent in normal mature T-cells and play a basic oncogenic role in T-cell leukemia if aberrantly activated [7, 22]. To investigate their impact in T-cell lymphomas we screened major entities of this cancer group. Analysis of angioimmunoblastic T-cell lymphoma (AITL), anaplastic large cell lymphoma (ALCL), adult T-cell leukemia/lymphoma (ATLL), HSTL, natural killer/T-cell lymphoma (NKTL) and PTCL was performed using public expression profiling datasets GSE6338, GSE19069, GSE57944, and GSE19067 (Supplementary Figures 1–4). Overall our screen identified 19 aberrantly overexpressed NKL homeobox genes in subsets of all analyzed T-cell lymphoma entities (Table 1). These genes comprised seven code-members (HHEX, HLX, MSX1, NANOG, NKX2-3, NKX3-1, NKX6-3) and 12 ectopically activated genes normally silent in hematopoiesis (DLX4, EN1, EN2, HMX2, NKX1-1, NKX2-1, NKX2-2, NKX2-5, NKX6-1, TLX1, TLX3, VAX2). The most widespread overexpressed NKL homeobox genes in T-cell lymphomas were HHEX, HLX, MSX1 and NKX2-3, all members of the NKL-code. The highest number of differentially overexpressed NKL homeobox genes was detected in PTCL and NKTL (*N* = 11) while ATLL and HSTL each showed the lowest number of deregulated genes (*N* = 6). Collectively, our data demonstrate that NKL homeobox gene deregulation is a frequent event in both, T-cell leukemia and T-cell lymphoma.

**Table 1:** Expression patterns of NKL homeobox genes in normal hematopoiesis and T-cell lymphomas

	Hematopoiesis											T-cell lymphomas					
Gene	HSC	LMPP	CLP	BCP	GCB	MBC	PC	NK	DN	DP	T	PTCL	AITL	ALCL	ATLL	HSTL	NKTL
**HHEX**	+	+	+	+	+	+			+			+	+	+	+	+	+
**HLX**	+	+	+	+					+			+	+	+	+	+	+
**MSX1**			+	+				+				+	+	+	+	+	
**NANOG**		+															+
**NKX2-3**	+											+	+	+	+	+	+
**NKX3-1**	+								+								+
**NKX6-3**				+			+										+
**TLX2**									+								
**VENTX**									+								
**DLX4**																	+
**EN1**												+					
**EN2**												+					
**HMX2**																	+
**NKX1-1**												+	+	+			
**NKX2-1**												+	+	+	+		
**NKX2-2**												+	+	+	+		
**NKX2-5**												+		+		+	+
**NKX6-1**												+		+			
**TLX1**																+	
**TLX3**																	+
**VAX2**																	+

The expression data for NKL homeobox genes in normal hematopoiesis are reported elsewhere [7, 8, 19], indicating physiological gene activities (+). Hematopoietic stem cell (HSC), lymphoid and myeloid primed progenitor (LMPP), common lymphoid progenitor (CLP), B-cell progenitor (BCP), memory B-cell (MBC), plasma cell (PC), NK-cell (NK), double negative T-cell (DN), double positive T-cell (DP), T-cell (T). The expression data of T-cell lymphomas indicate overexpressed NKL homeobox genes (+).

### MSX1 overexpression in HSTL

In the following, we focused our study on MSX1. This NKL homeobox gene is physiologically expressed in early immature lymphocytes including CLPs and B-cell progenitors in addition to mature NK-cells but downregulated in terminal development of B- and T-cells [7, 8, 19]. Accordingly, MSX1 represents an oncogene in T-ALL and a tumor suppressor in NK-cell leukemia [12, 19]. Consistently, MSX1 was not found to be overexpressed in NKTL in contrast to remaining T-cell lymphoma entities (Table 1). To search for a T-cell lymphoma cell line model overexpressing MSX1 we screened dataset GSE57083 (GEO) which contains expression profiling data of 123 hematopoietic cell lines. This screening revealed HSTL-derived cell lines DERL-2 (Supplementary Figure 5).

**Figure 1:**
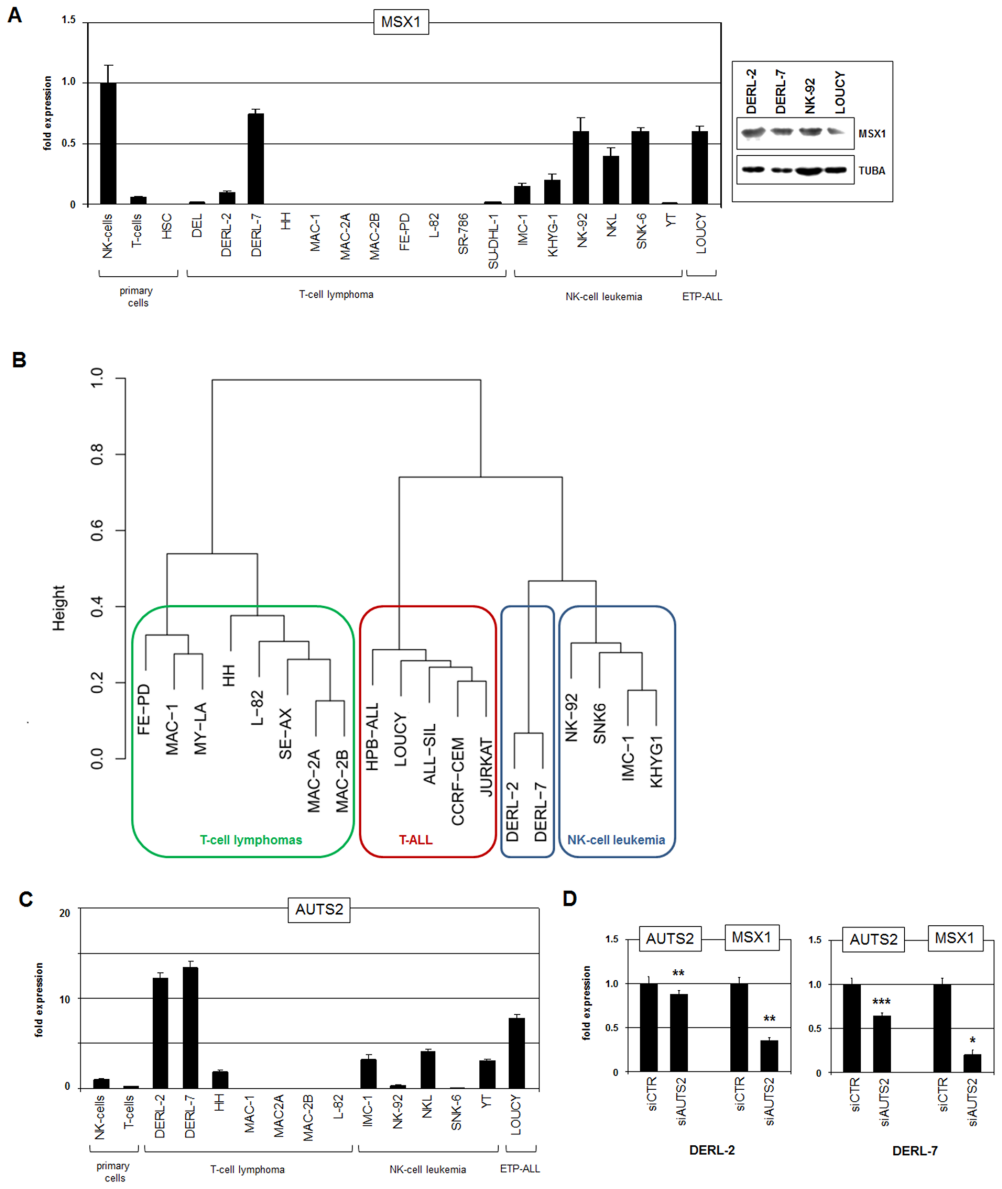
Gene expression analyses. (**A**) RQ-PCR analysis of MSX1 in primary cells and selected cell lines (left). Western blot analysis of MSX1 in selected cell lines (right). Alpha-tubulin (TUBA) served as loading control. (**B**) Gene expression profiling data of selected cell lines were used to construct a dendrogram. Of note, HSTL cell lines DERL-2 and DERL-7 cluster next to NK-cell leukemia cell lines while T-cell lymphoma and T-ALL cell lines built separated groups. (**C**) RQ-PCR analysis of AUTS2 in primary cells and selected cell lines. (**D**) RQ-PCR analysis for AUTS2 and MSX1 of DERL-2 (left) and DERL-7 (right) cells treated for siRNA-mediated knockdown of AUTS2.

Subsequent RQ-PCR analysis performed in selected T-cell lymphoma cell lines in comparison to samples of primary NK-cells, T-cells and HSCs confirmed aberrant overexpression of MSX1 in DERL-2 and demonstrated even higher levels in sister cell line DERL-7 (Figure 1A). In DERL-7 the expression level of MSX1 matched normal NK-cells while the lower expression of DERL-2 matched most leukemic NK-cell lines. Western blot analysis confirmed MSX1 expression at the protein level in DERL-2/7 and additionally in NK-92 and LOUCY (Figure 1A), indicating a likely functional relevance in these cells. HSTL-derived sister cell lines DERL-2 and DERL-7 were respectively established from peripheral blood and bone marrow of the same patient. They represent T-cells (rearranged TCR-genes, CD7+) but show in addition NK-cell characteristics (CD2+, CD56+) [23, 24]. Together, DERL-2 and DERL-7 expressed enhanced levels of MSX1 as observed in subsets of HSTL patients and, thus, represent suitable tools to analyze factors operating upstream in this type of T-cell lymphoma.

Cluster analysis using gene expression profiling data of cell lines derived from T-cell lymphomas, T-ALL, and NK-cell leukemia revealed the greater proximity of DERL-2/7 to NK-cells than to T-cells (Figure 1B). This finding underlined that HSTL-derived cell lines aberrantly show NK-cell characteristics as previously reported in primary HSTL cells [24, 25]. We speculated that this phenotype might be supported by MSX1 which is normally operating in mature NK-cells [19]. Therefore, we subsequently analyzed additional NK-cell specific factors including AUTS2. This chromatin-modulator converts polycomb repressor subtype PRC1.5 into an activator and plays a role in (de)regulation of NKL homeobox gene MSX1 in T-cells and NK-cells [15, 18, 19]. RQ-PCR analysis of AUTS2 in T-cell lymphoma cell lines and primary hematopoietic cells showed enhanced expression in DERL-2/7 (Figure 1C). Moreover, siRNA-mediated knockdown of AUTS2 in DERL-2 and DERL-7 resulted in reduced MSX1 expression in both cell lines (Figure 1D), demonstrating that this chromatin-factor also plays an activating role in this type of T-cell malignancy.

ETS1, ID2 and TBX21/T-bet represent master regulators of NK-cell development [3]. To analyze these genes we quantified their transcripts in cell lines and primary hematopoietic cells by RQ-PCR (Supplementary Figure 6A). The expression levels of all three genes were similar in DERL-2 and DERL-7, thus resembling leukemic NK-cell lines. These results affirmed the NK-cell character of DERL-2/7 indicated by the clustering and cell line data reported previously [24]. Of note, siRNA-mediated knockdown of AUTS2 or MSX1 in DERL-2/7 did not consistently alter the expression levels of ETS1, ID2 or TBX21 (Supplementary Figure 6B), suggesting just marginal impact. Furthermore, knockdown of ETS1, ID2 or TBX21 did not alter the expression level of MSX1 (Supplementary Figure 6C), excluding mutual regulatory connections. Taken together, HSTL-derived cell lines DERL-2/7 showed aberrant NK-cell-like expression patterns, including activities of NKL homeobox gene MSX1 and its activator AUTS2. These findings prompted a search for additional mechanisms and factors/genes involved in the (de)regulation of MSX1 in DERL-2/7 cells and HSTL patients as described below.

### Chromosomal and genomic analyses of DERL-2 and DERL-7

**Figure 2:**
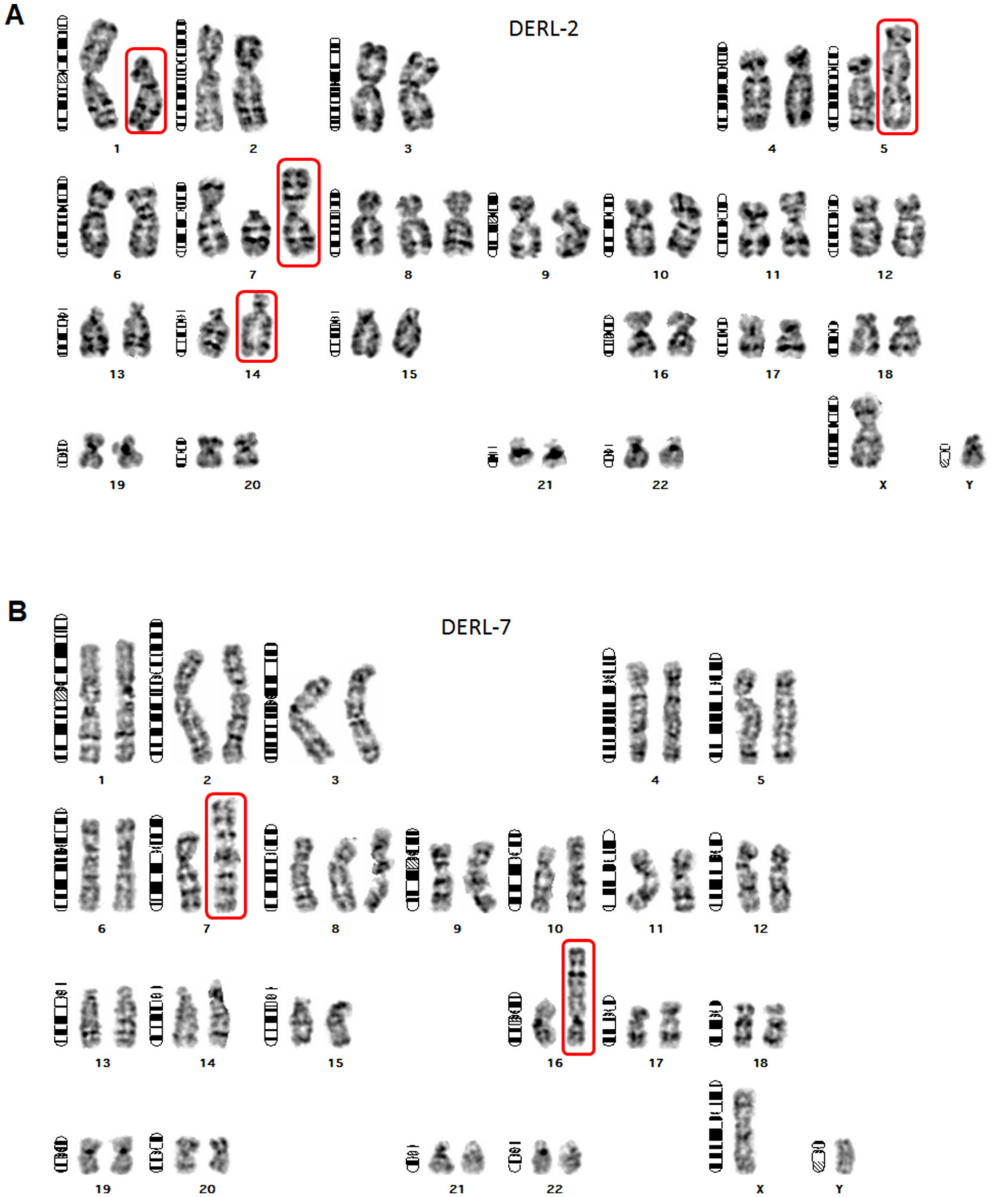
Karyotypes of DERL-2 and DERL-7. Giemsa-stained metaphase chromosomes of HSTL-derived cell lines DERL-2 (**A**) and DERL-7 (**B**) were used to deduce their karyotypes as follows. DERL-2: 48(46-49)<2n>XY,t(1;5)(p13;q32),+7,i(7)(q10-11),+8,add(10)(p11),dup(14)(q21q??); DERL-7: 47(46-47)<2n>XY,i(7)(q10-11),t(7;16)(q11;p13),+8,add(10)(p12.1). Red boxes highlight abnormal chromosomes analyzed in this study.

In T-ALL aberrant expression of NKL homeobox genes is frequently associated with their chromosomal/genomic rearrangement [11]. To examine if this mechanism plays a role for MSX1 in DERL-2/7 as well we performed karyotyping of both cell lines. These analyses revealed i(7q) in DERL-2 and DERL-7, t(1;5)(p13;q32) and dup(14q) exclusively in DERL-2, and der(7)t(7;16)(q11;p13) exclusively in DERL-7. Neither cell line bore rearrangements of chromosome 4 which hosts the MSX1 gene at 4p16 (Figure 2).

**Figure 3:**
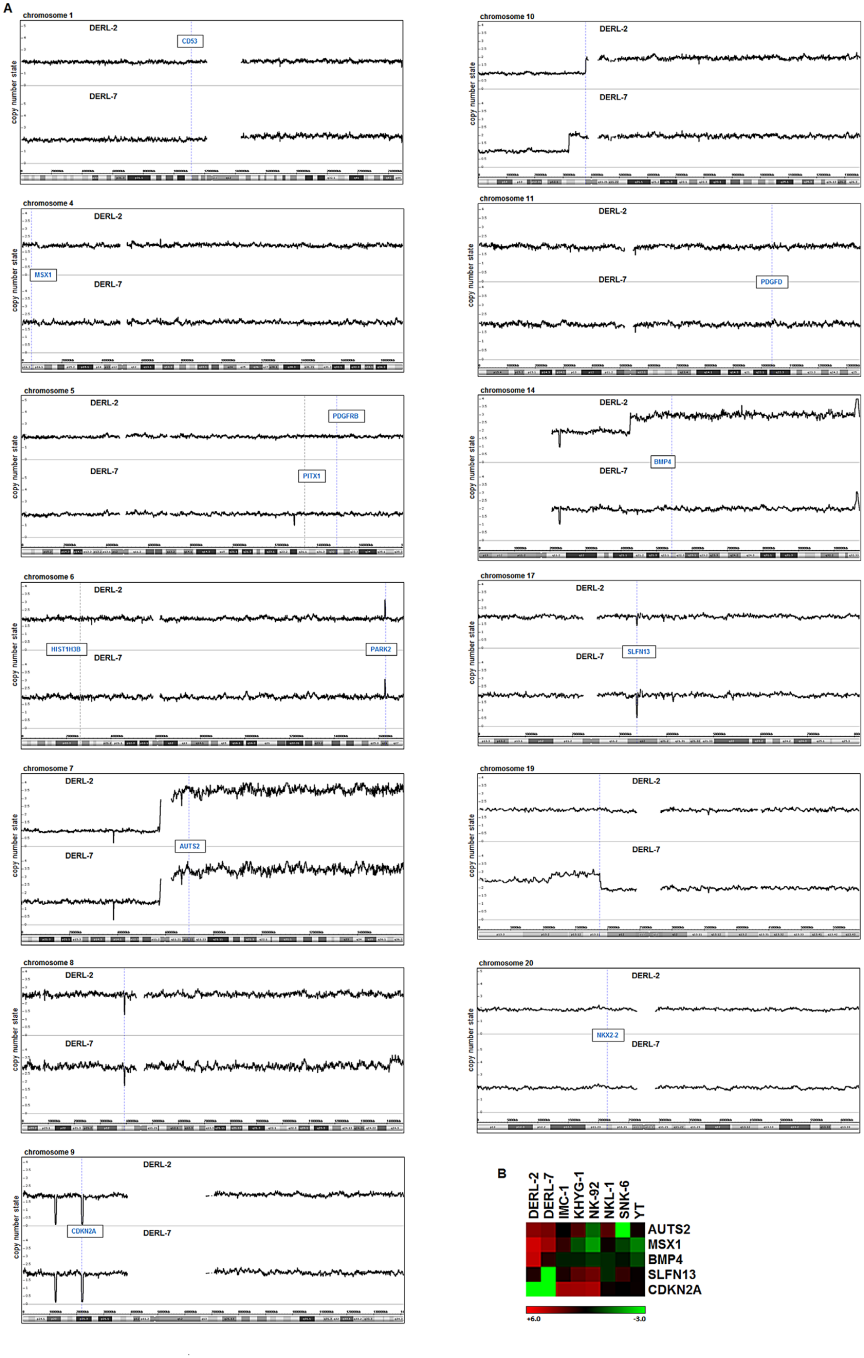
Genomic profiling of DERL-2 and DERL-7. (**A**) Genomic profiling data of HSTL cell lines DERL-2 and DERL-7 showing selected chromosomes. Selected gene loci are indicated in boxes. (**B**) Heatmap obtained from expression profiling data of DERL-2/7 and six NK-cell lines for AUTS2, MSX1, BMP4, SLFN13 and CDKN2A.

Genomic profiling identified several copy-number alterations in DERL-2/7. However, this analysis confirmed wild type configurations of the MSX1 locus in both cell lines (Figure 3A). In contrast, MSX1 activator AUTS2 is located at 7q11 and showed copy-number gains caused by the generation of der(7)t(7;16) in DERL-7, and of i(7q) in both cell lines. The latter abnormality is a hallmark of HSTL [26], and may thus underlie elevated expression levels of AUTS2 und its regulatory target MSX1. Additionally, both cell lines harbor overlapping terminal deletions of the short arm of chromosome 10. The mapping coordinates of this deletion differed between DERL-2 and DERL-7, indicating their independent origin/evolution. Similarly, a chromosomal duplication at 19p13 was detectible in DERL-7 only. Finally, copy-number alterations showing corresponding deregulation of particular gene activities included BMP4 at 14q22, CDKN2A at 9p21, and SLFN13 at 17q12 (Figure 3A, 3B).

Whole genome sequencing of DERL-2 and DERL-7 allowed the identification of both, chromosomal rearrangements and gene mutations. However, while these data confirmed the absence of rearrangements at MSX1 they revealed mutations in the genes HIST1H3B(K27M), KDM7A(P897L), SETD2(P1962L), STAT5B(N642H) and TLE1(P287A) in both cell lines. These mutations were confirmed by Sanger-sequencing (Supplementary Figure 7) and match exome-sequencing data obtained from DERL-2/7 and HSTL patients [27]. But we detected no AUTS2 mutations as we have recently described in NK-cell leukemia cell lines [19]. Taken together, our chromosomal/genomic data excluded rearrangements at the MSX1 locus but revealed specific copy-number alterations and rearrangements which might contribute indirectly to MSX1 deregulation.

### DERL-2 contains fusion gene CD53-PDGFRB

**Figure 4:**
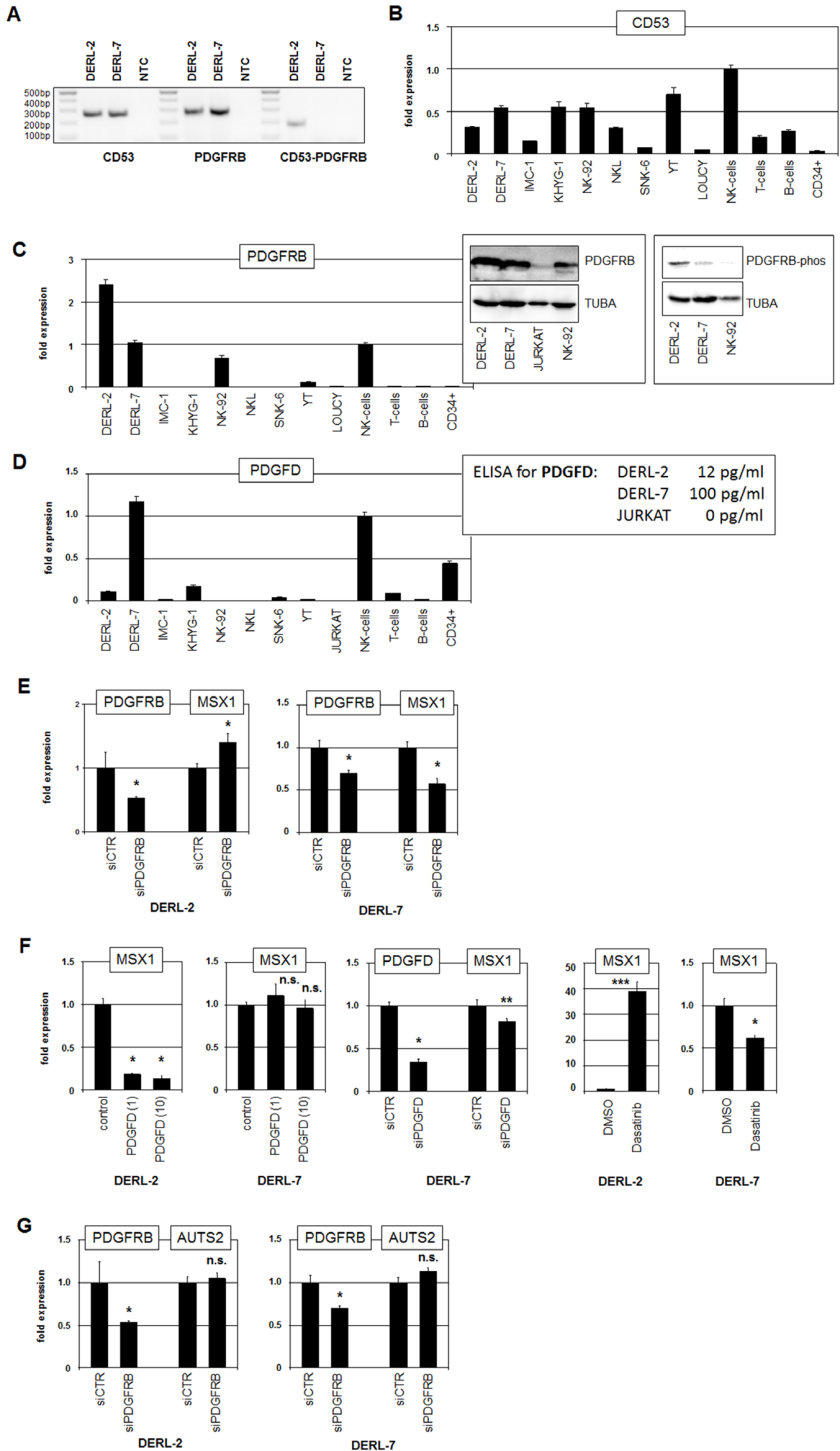
Analyses of fusion gene CD53-PDGFRB. (**A**) RT-PCR analysis of CD53, PDGFRB and CD53-PDGFRB fusion transcripts in cell lines DERL-2 and DERL-7. NTC: no template control. (**B**) RQ-PCR analysis of CD53 in selected cell lines and primary cells. (**C**) RQ-PCR analysis of PDGFRB in selected cell lines and primary cells (left). Western blot analysis of PDGFRB (middle) and phospho-PDGFRB (right) was performed in selected cell lines. TUBA served as loading control. (**D**) RQ-PCR analysis of PDGFD in selected cell lines and primary cells (left). Data for PDGFD protein levels in the supernatants as obtained by ELISA are indicated (right). (**E**) RQ-PCR analysis for PDGFRB and MSX1 of DERL-2 cells (left) and DERL-7 cells (right) treated for siRNA-mediated knockdown of PDGFRB. (**F**) RQ-PCR analysis of MSX1 in DERL-2 cells and DERL-7 cells treated with 1 ng/ml and 10 ng/ml recombinant PDGFD (left). RQ-PCR analysis for PDGFD and MSX1 of DERL-7 cells treated for siRNA-mediated knockdown of PDGFD (middle). RQ-PCR analysis for MSX1 of DERL-2 and DERL-7 cells treated with PDGFR kinase-inhibitor Dasatinib (right). (**G**) RQ-PCR analysis for PDGFRB and AUTS2 of DERL-2 cells (above) and DERL-7 cells (below) treated for siRNA-mediated knockdown of PDGFRB.

Karyotyping revealed novel chromosomal rearrangement, t(1;5)(p13;q32), exclusive to DERL-2. Genome sequencing showed that this translocation resulted in the fusion of CD53 at 1p13 to PDGFRB at 5q32 (Supplementary Figure 8). The breakpoints were located in intron 2 of CD53 and intron 11 of PDGFRB. Expression of the resultant fusion gene CD53-PDGFRB was confirmed by RT-PCR (Figure 4A). RQ-PCR analysis of the fusion partners demonstrated reduced expression of CD53 and elevated expression of PDGFRB in DERL-2 as compared to DERL-7 (Figure 4B, 4C). Thus, the regulatory upstream region of CD53 plausibly drives the expression of PDGFRB in DERL-2. Western blot analysis showed strong PDGFRB expression at the protein level in DERL-2 and DERL-7 and indicated no significant difference between both cell lines (Figure 4C). In contrast, Western blot analysis of activated phospho-PDGFRB demonstrated a strong signal in DERL-2 and a weak signal in DERL-7 while NK-92 showed no signal at all (Figure 4C), indicating intrinsic differences of the activities of this pathway in DERL-2 and DERL-7.

The fusion-transcript consists of two CD53 exons at the 5′-end and of twelve PDGFRB exons at the 3′-end which lacks the encoded ligand-binding domain but retains the tyrosine-kinase domain. This aberrant configuration of PDGFRB has been detected in combination with other fusion partners in myeloid malignancies supporting an oncogenic function for this receptor gene [28]. Of note, DERL-2 contains in addition to the PDGFRB fusion gene one PDGFRB wild type allele. Consideration of expression profiling data of DERL-2/7 for genes encoding known PDGFR-ligands revealed significant levels of PDGFD only (data not shown). RQ-PCR analysis of PDGFD encoding RNA showed enhanced levels in fusion-negative DERL-7 cells (Figure 4D). These RNA expression levels correlated with secreted PDGFD protein levels as quantified by ELISA in the supernatants from the cell lines (Figure 4D). Therefore, autocrine activation of PDGFRB-signaling by PDGFD may play a role in DERL-7 but not in DERL-2. Interestingly, normal NK-cells expressed PDGFRB and PDGFD at similar RNA levels as detected in DERL-7 (Figure 4C), indicating that this pathway performs activities important for normal NK-cell function.

To investigate whether PDGFRB is involved in MSX1 regulation in HSTL we performed siRNA-mediated knockdown experiments targeting sequences located in exon 17 which are present in both wild type PDGFRB and the fusion-transcript. Interestingly, reduction of PDGFRB resulted in elevated MSX1 transcript levels in DERL-2 while this procedure reduced MSX1 expression in DERL-7 (Figure 4E). Treatment of the cell lines with recombinant PDGFD resulted in repression of MSX1 in PDGFD-low expressing DERL-2 but showed no significant effect in PDGFD-high expressing DERL-7 cells (Figure 4F). However, siRNA-mediated knockdown of PDGFD in DERL-7 resulted in reduced MSX1 expression (Figure 4F). These data indicated that ligand-induced PDGFRB-signaling contrastingly inhibits MSX1 expression in DERL-2 but activates this gene in DERL-7. This conclusion was further confirmed by treatment of DERL-2/7 with PDGFR kinase-inhibitor Dasatinib which resulted in MSX1 activation in DERL-2 and MSX1 repression in DERL-7 (Figure 4F). Of note, the expression of MSX1-activating AUTS2 was not impacted by knockdown of PDGFRB in either cell line, discounting this regulatory connection (Figure 4G). Taken together, we identified a novel chromosomal rearrangement in DERL-2 which resulted in the fusion of CD53 and PDGFRB. We further demonstrated that this fusion gene inhibited MSX1 expression while signaling via the normal receptor activated MSX1. This functional difference corresponds to the differential MSX1 transcript levels in the cell lines DERL-2 and DERL-7.

### Aberrant chromatin conformation activates MSX1 in DERL-2/7

**Figure 5:**
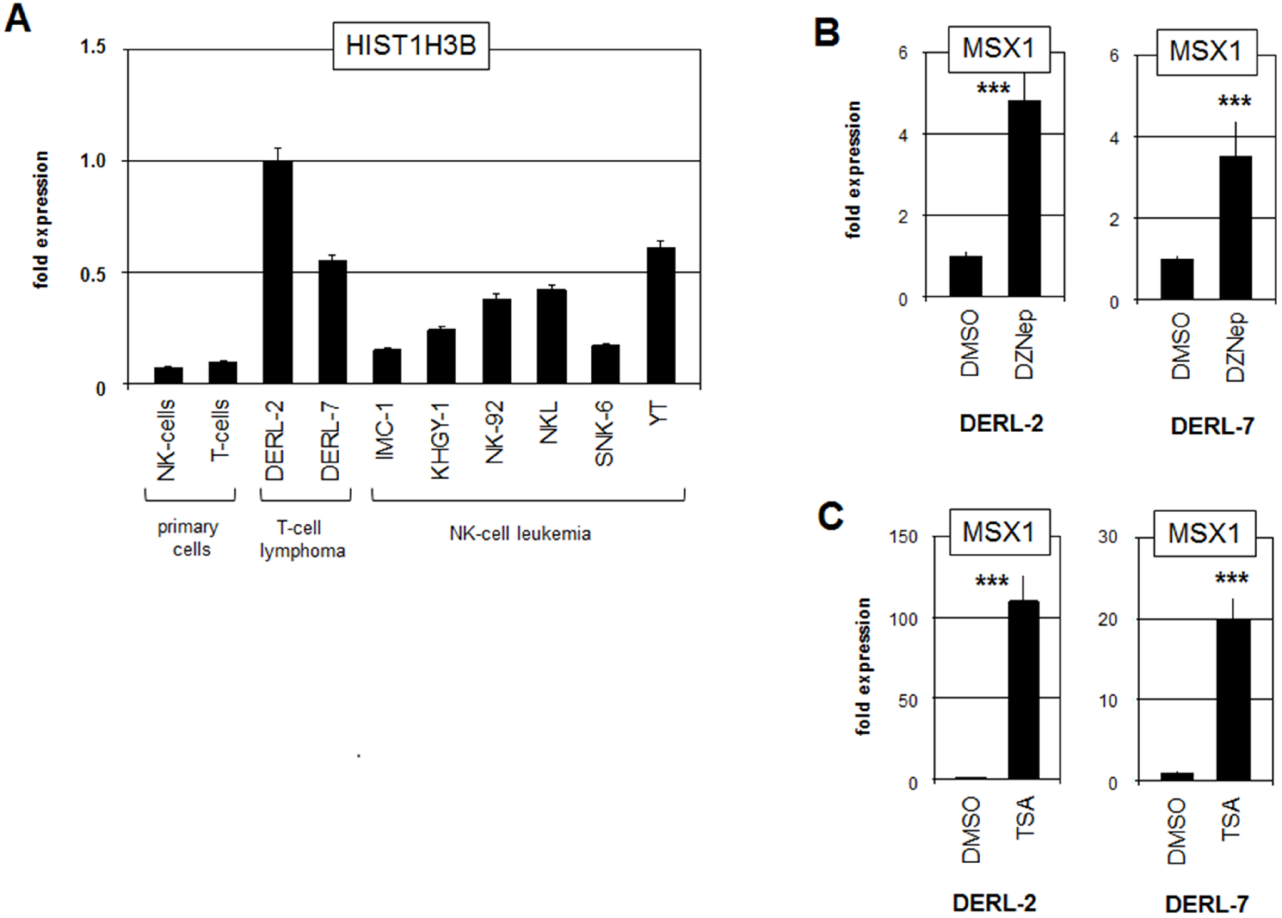
Analysis of mutated histone HIST1H3B. (**A**) RQ-PCR analysis of HIST1H3B in primary cells and selected cell lines. (**B**) RQ-PCR analysis of MSX1 in DERL-2 (left) and DERL-7 (right) treated with EZH2-inhibitor DZNep. (**C**) RQ-PCR analysis of MSX1 in DERL-2 (left) and DERL-7 (right) treated with histone deacetylase-inhibitor TSA.

Aberrant expression levels of histones and chromatin-modifiers have been reported to deregulate the activity of NKL homeobox genes in T-ALL and B-cell lymphomas, highlighting the significance of this deregulatory mechanism [14, 15]. Therefore, we examined the role of the mutated histone HIST1H3B(K27M) which may regulate the activity of the MSX1 gene via altered chromatin conformation. RQ-PCR analysis of HIST1H3B in cell lines and primary samples of NK- and T-cells demonstrated elevated expression levels in DERL-2/7 cells (Figure 5A), implying an oncogenic role for this gene. The identified mutation in HIST1H3B prevents suppressive methylation at residue K27 by EZH2 [29]. To analyse the impact of H3K27-methylation on MSX1 expression we treated DERL-2 and DERL-7 cells with EZH2-inhibitor DZNep (Figure 5B). This exercise resulted in increased levels of MSX1 and showed that EZH2 mediated repression of MSX1 in both cell lines. Thus, elevated levels of mutated histone HIST1H3B(K27M) may contribute to MSX1 activation via inhibition of EZH2. Treatment of DERL-2/7 with histone deacetylase inhibitor TSA also resulted in increased MSX1 expression (Figure 5C), supporting the role of histone modifications in MSX1 gene activity. Collectively, these data highlight the regulatory role of chromatin by demonstrating that reduced histone H3-trimethylation at position K27 is involved in the aberrant activation of MSX1 in HSTL, just like overexpressed chromatin modulator AUTS2 which mediates activating histone acetylation [18].

### Comparative expression profiling of HSTL cell lines and patients

To identify additional MSX1-regulators in HSTL we performed comparative expression profiling analyses. First, we compared DERL-2 (MSX1 low) and DERL-7 (MSX1 high) to reveal specific differences among the top-1000 differentially expressed genes: DERL-2 expressed enhanced levels of BMP4, CD8A and SLFN13 while DERL-7 expressed CD3D, CD3E, PDGFD and TLE1 at higher levels (Supplementary Table 1). Next, we compared DERL-7 (MSX1 high) and three NK-cell lines (MSX1 low: IMC1, KHYG1, YT), demonstrating in DERL-7 higher levels of NKX2-2, OTX2, PDGFD, PITX1, SIX1 and TLE1 and lower levels of CDKN2A, SLFN13 and STAT5B (Supplementary Table 2). For comparison of HSTL patients we analyzed dataset GSE57944 (including two MSX1-high and two MSX1-low samples) and used an associated R-based online-tool at GEO (https://www.ncbi.nlm.nih.gov). This comparison revealed a significant correlation of PDGFRA with MSX1 expression (Supplementary Figure 9). Finally, we used dataset GSE19067 to compare four HSTL patients (MSX1-high) with seven NKTL patients (MSX1-low). This analysis revealed elevated expression levels of NKX2-3, TLX1, BAMBI and ENG in MSX1-high HSTL patients (Supplementary Figure 10). Taken together, these data indicated four categories of gene candidates probably involved in MSX1 (de)regulation: (1) PDGF-signalling pathway (PDGFD, PDGFRA), (2) BMP-signalling pathway (BAMBI, BMP4, ENG), (3) homeobox genes (NKX2-2, OTX2, PITX1 and SIX1), and (4) TLE corepressors (TLE1). Interestingly, most of these candidate genes have been previously reported to deregulate MSX1 expression in lymphoid malignancies [12, 30–32], suggesting that they might play a similar role in HSTL as well. Accordingly, we decided to focus attention on the deregulating potentials of the BMP-receptor ligand BMP4 and of the homeobox genes NKX2-2 and PITX1.

### BMP4, NKX2-2 and PITX1 regulate MSX1 in HSTL

**Figure 6:**
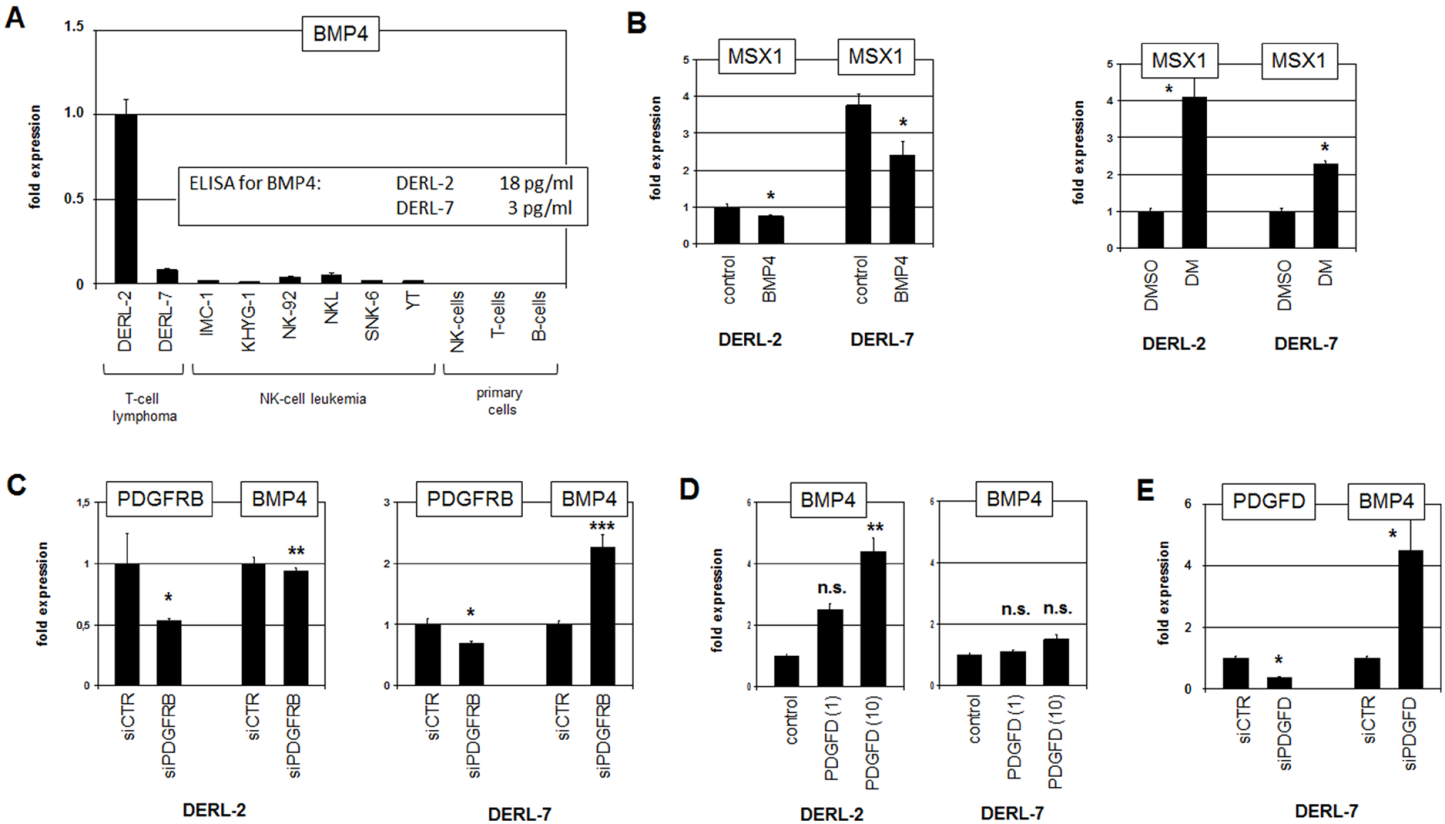
Analysis of BMP4. (**A**) RQ-PCR analysis of BMP4 in selected cell lines and primary cells. Data for BMP4 protein levels in the supernatants as obtained by ELISA are indicated (insert). (**B**) RQ-PCR analysis of MSX1 in DERL-2 and DERL-7 cells treated with recombinant BMP4 (left) and with dorsomorphin (DM) (right). (**C**) RQ-PCR analysis for PDGFRB and BMP4 of DERL-2 cells (left) and DERL-7 cells (right) treated for siRNA-mediated knockdown of PDGFRB. (**D**) RQ-PCR analysis of BMP4 in DERL-2 (left) and DERL-7 (right) treated with 1 ng/ml or 10 ng/ml recombinant PDGFD. (**E**) RQ-PCR analysis for PDGFD and BMP4 of DERL-7 cells treated for siRNA-mediated knockdown of PDGFD.

RQ-PCR analysis of BMP4 in T-cell lymphoma cell lines and normal primary hematopoietic cells confirmed strong overexpression in DERL-2 (Figure 6A). Of note, elevated BMP4 expression correlated with its copy-number gain at 14q22 in this cell line which may underlie its aberrant activation (Figure 3A). Treatment of DERL-2 and DERL-7 with recombinant BMP4 protein resulted in weak and strong downregulation of MSX1, respectively (Figure 6B). Consistently, treatment of both cell lines with BMP-receptor inhibitor dorsomorphin mediated enhanced MSX1 expression (Figure 6B), supporting suppression of MSX1 transcription via BMP-signalling. SiRNA-mediated downregulation of PDGFRB resulted in strong upregulation of BMP4 expression in DERL-7 and slight downregulation in DERL-2 (Figure 6C). Treatment of DERL-2 and DERL-7 with PDGFD enhanced BMP4 transcription in PDGFD-low expressing DERL-2 but showed no effect in PDGFD-high expressing DERL-7 cells (Figure 6D). In contrast, siRNA-mediated knockdown of PDGFD in DERL-7 resulted in elevated BMP4 expression (Figure 6E). Collectively, these results demonstrated that PDGFRB-signalling activates BMP4 in DERL-2 while repressing this gene in DERL-7, indicating fundamental differences in downstream activities of this pathway.

**Figure 7:**
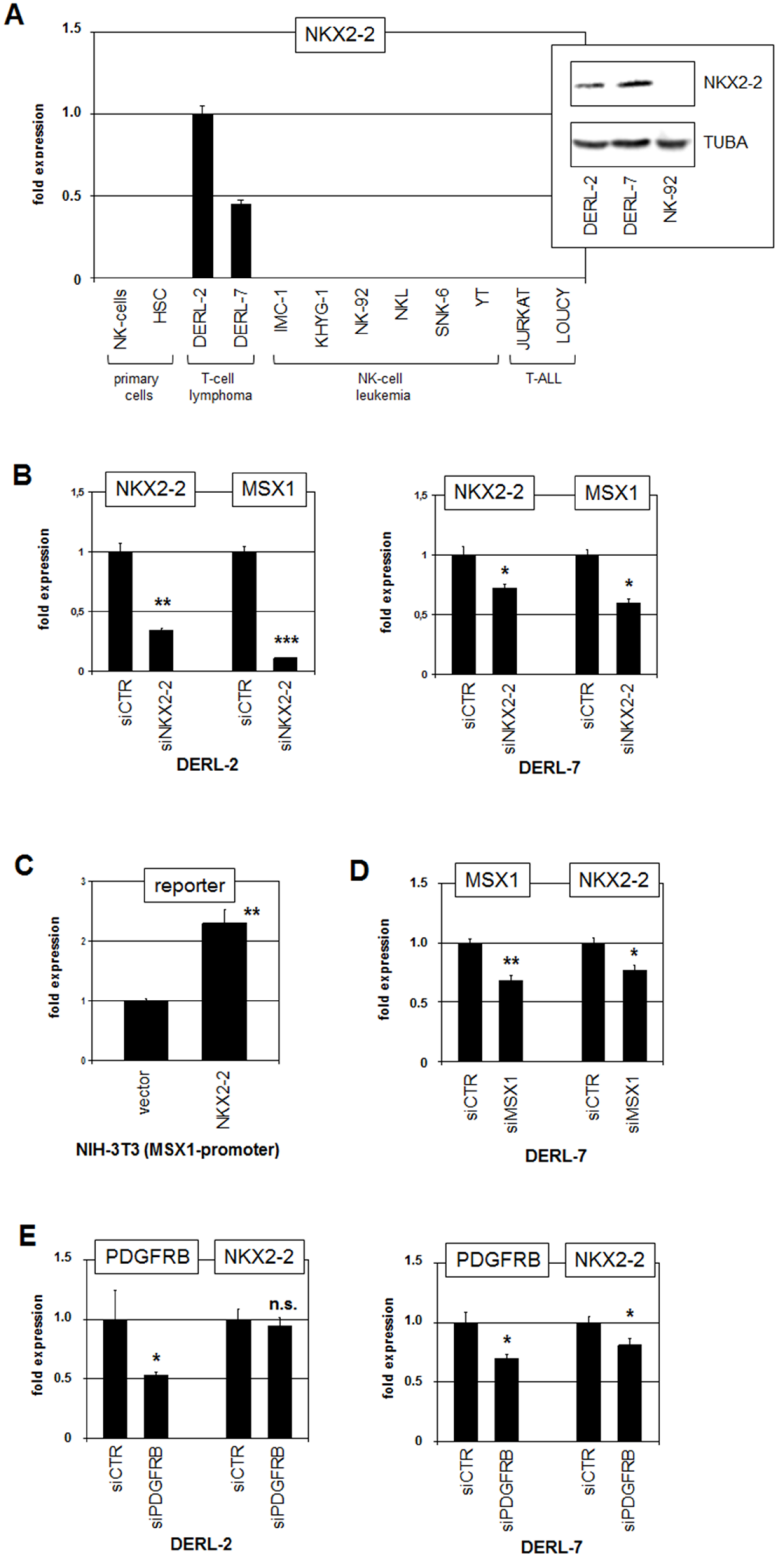
Analysis of NKX2-2. (**A**) RQ-PCR analysis of NKX2-2 in primary cells and selected cell lines. Western blot analysis of NKX2-2 in selected cell lines (insert). TUBA served as loading control. (**B**) RQ-PCR analysis for NKX2-2 and MSX1 of DERL-2 cells (left) and DERL-7 cells (right) treated for siRNA-mediated knockdown of NKX2-2. (**C**) Reporter gene assay was performed in NIH-3T3 cells using a promoter fragment of MSX1. Forced expression of NKX2-2 resulted in increased reporter gene activity. (**D**) RQ-PCR analysis for MSX1 and NKX2-2 of DERL-7 cells treated for siRNA-mediated knockdown of NKX2-2. (**E**) RQ-PCR analysis for PDGFRB and NKX2-2 of DERL-2 cells (left) and DERL-7 cells (right) treated for siRNA-mediated knockdown of PDGFRB.

RQ-PCR analysis of NKX2-2 in normal primary cells and malignant cell lines confirmed aberrant NKX2-2 expression in DERL-2 and DERL-7 (Figure 7A). Western blot analysis demonstrated significant NKX2-2 expression in the cell lines at the protein level as well. SiRNA-mediated knockdown of NKX2-2 resulted in reduced expression of MSX1 in both, DERL-2 and DERL-7 (Figure 7B), showing that NKX2-2 activates MSX1 transcription. Promoter analysis of MSX1 revealed a potential binding site for NKX2-2 (CCACTT) at -656 bp. Subsequent performance of a reporter gene assay confirmed direct activation of MSX1 transcription by NKX2-2 via this site (Figure 7C). SiRNA-mediated knockdown of MSX1 in DERL-7 resulted in reduced expression of NKX2-2 (Figure 7D), demonstrating reciprocal activation of these NKL homeobox genes. Genomic copy-number analysis excluded any gain at the NKX2-2 loci at 20p11 in DERL-2/7 (Figure 3A), discounting this potential mechanism for aberrant activation. SiRNA-mediated knockdown of PDGFRB showed no significant alteration in NKX2-2 expression in DERL-2 but slightly reduced levels in DERL-7 (Figure 7E), indicating that NKX2-2 is a downstream target of this signalling pathway in DERL-7 only.

**Figure 8:**
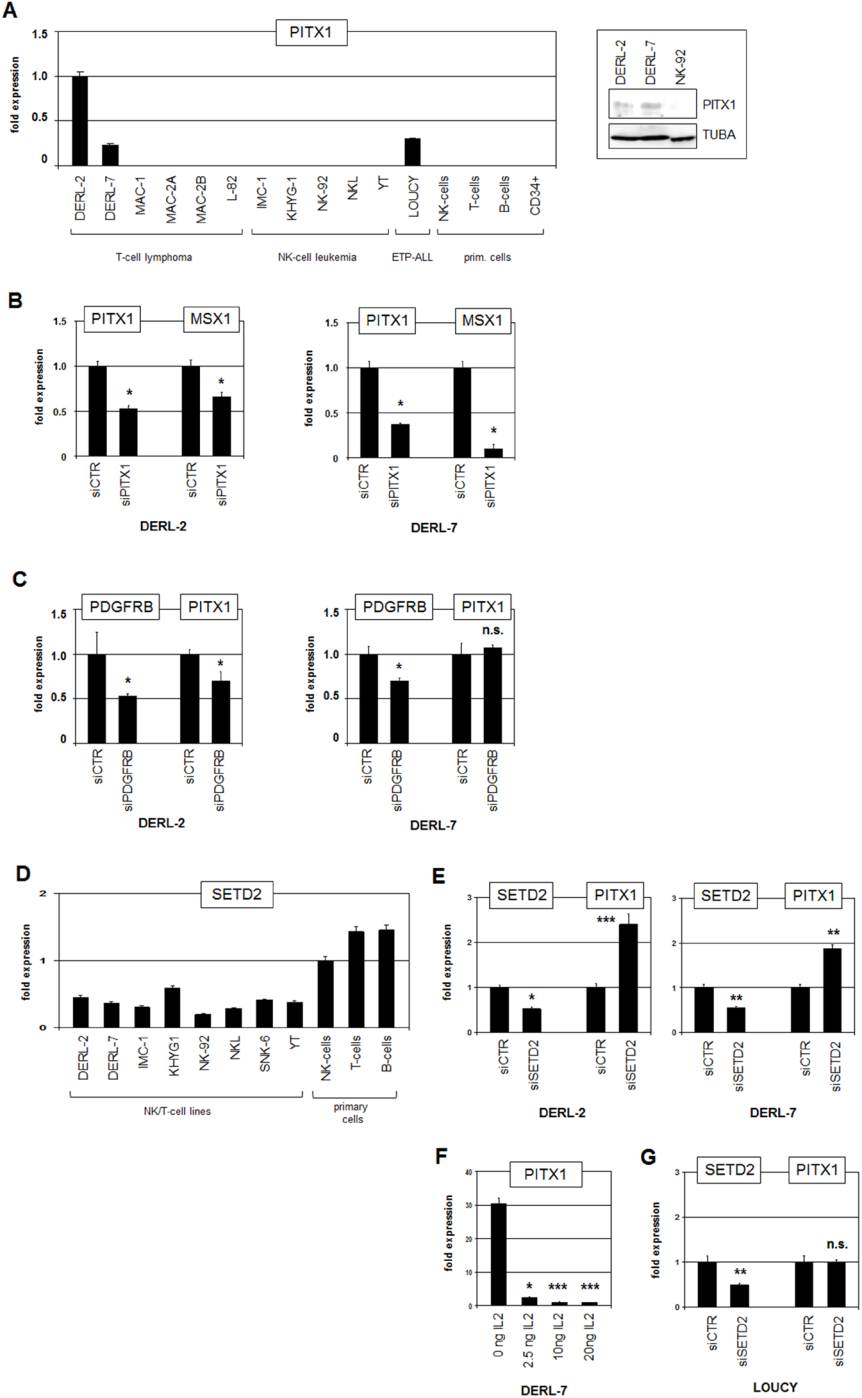
Analysis of PITX1 and SETD2. (**A**) RQ-PCR analysis of PITX1 in selected cell lines and primary cells (left). Western blot analysis of PITX1 in selected cell lines (right). TUBA served as loading control. (**B**) RQ-PCR analysis for PITX1 and MSX1 of DERL-2 (left) and DERL-7 (right) treated for siRNA-mediated knockdown of PITX1. (**C**) RQ-PCR analysis for PDGFRB and PITX1 of DERL-2 (left) and DERL-7 (right) treated for siRNA-mediated knockdown of PDGFRB. (**D**) RQ-PCR analysis of SETD2 in selected cell lines and primary cells. (**E**) RQ-PCR analysis for SETD2 and PITX1 of DERL-2 (left) and DERL-7 (right) treated for siRNA-mediated knockdown of SETD2. (**F**) RQ-PCR analysis of PITX1 in DERL-7 cells treated with indicated amounts of recombinant IL2. (**G**) RQ-PCR analysis for SETD2 and PITX1 of LOUCY cells treated for siRNA-mediated knockdown of SETD2.

RQ-PCR analysis of PITX1 in cell lines and normal primary cells confirmed strong ectopic overexpression in DERL-2 (Figure 8A). Of note, DERL-7 also expressed PITX1 at high levels, resembling those quantified in T-ALL cell line LOUCY which was reported to carry an activating deletion in the regulatory downstream region of this gene [31]. However, this deletion was absent in DERL-2 and DERL-7 (Supplementary Figure 11). Furthermore, neither DERL-2 nor DERL-7 contain copy-number gains at their PITX1 loci at 5q31 (Figure 3A), discounting gene dosage as mechanism of gene deregulation. Western blot analysis confirmed PITX1 protein expression and showed similar levels in both cell lines (Figure 8A), indicating functional potential for PITX1. SiRNA-mediated knockdown of PITX1 in DERL-2 and DERL-7 caused reduced MSX1 expression levels (Figure 8B), suggesting that PITX1 activated MSX1 in both cell lines. SiRNA-mediated knockdown of PDGFRB resulted in reduced expression of PITX1 in DERL- 2 but showed no effect in DERL-7 (Figure 8C). Thus, PDGFRB-signalling activates PITX1 in DERL-2 only which consistently expressed higher PITX1 transcript levels than DERL-7.

PITX1 expression is inhibited by IL2/STAT1-signalling in T-ALL cells [31]. SETD2 has been reported to activate STAT1 and is reportedly mutated in HSTL patients and DERL-2/7 [27, 33]. RQ-PCR analysis of SETD2 in cell lines and normal primary cells showed reduced expression levels in DERL-2/7 (Figure 8D), indicating tumor suppressor activity [27]. SiRNA-mediated knockdown of SETD2 produced elevated PITX1 expression levels (Figure 8E), demonstrating that SETD2 inhibits PITX1 transcription probably via STAT1-activation. Consistently, treatment of DERL-7 with STAT1-activator IL2 effected reduced PITX1 expression (Figure 8F). Moreover, siRNA-mediated knockdown of SETD2 showed no effect on PITX1 expression in LOUCY cells which lack the repressive STAT1 binding site due to a chromosomal deletion [31] (Figure 8G). Thus, STAT1 inhibits the expression of PITX1 in cooperation with IL2 and SETD2. However, this regulatory mechanism is disturbed in DERL-2/7 and subsequent aberrant PITX1 activity supports the expression of MSX1. In conclusion, our data show that several factors including specific chromatin components, PDGF- and BMP-signalling pathways, and homeodomain TFs NKX2-2 and PITX1 conspire to (de)regulate NKL homeobox gene MSX1 in HSTL. Aberrant MSX1 activity may contribute to disturbed T-/NK-cell differentiation in this malignancy.

## DISCUSSION

Deregulated NKL homeobox genes represent a frequent abnormality in T-ALL counting 24 genes to date [7, 10]. Indeed, TLX1 the first subclass member described as an oncogene in this malignancy still serves as an important prognostic marker [9, 34]. Detailed downstream activities of TLX1 and other subclass members have been discovered and illuminate their oncogenic roles in T-ALL [13, 22, 35–38]. The structural similarity of this group of TFs suggests analogous oncogenic functions although differences have been described. In this study, we identified 19 deregulated NKL homeobox genes in six T-cell lymphoma entities comprising AITL, ALCL, ATLL, HSTL, NKTL and PTCL (Table 1). These findings suggest that this homeobox gene subclass plays an oncogenic role across all types of T-cell malignancy.

**Figure 9:**
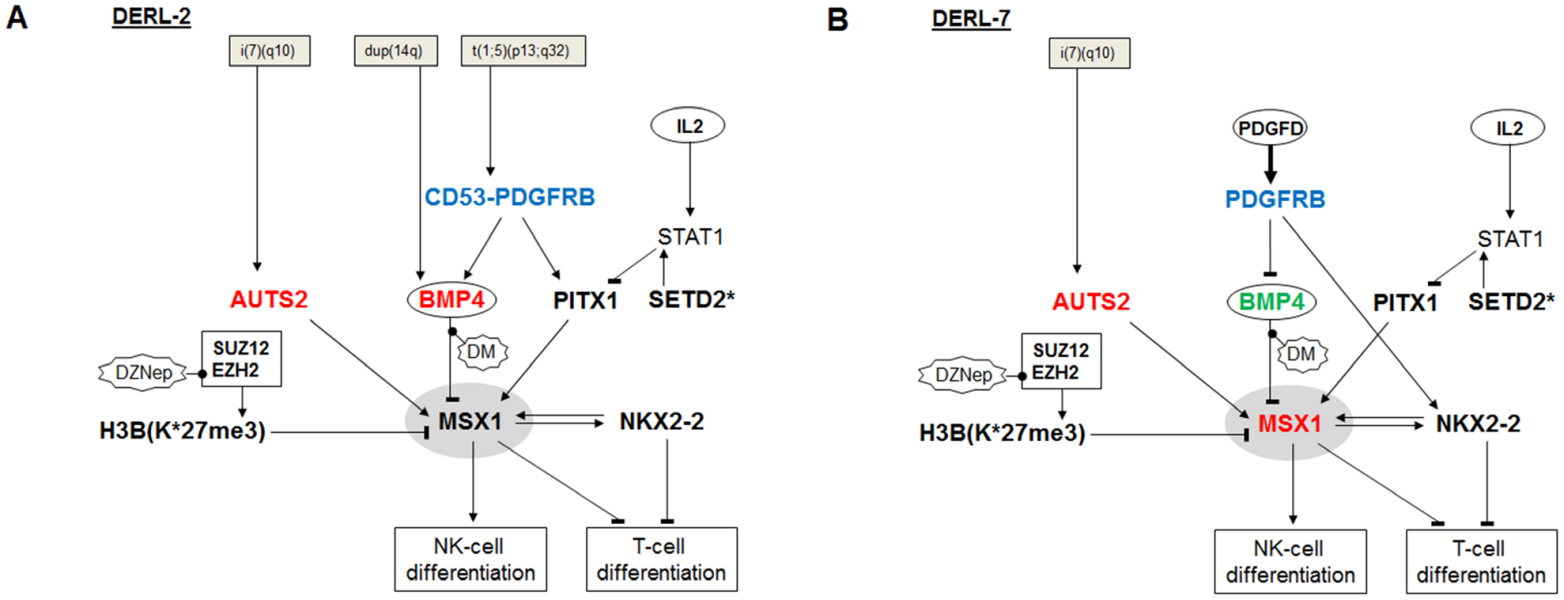
Aberrant gene regulatory networks in HSTL cell lines. Upstream regulators of MSX1 in (**A**) DERL-2 and (**B**) DERL-7 generate different levels of MSX1 expression. Asterisks (^*^) indicate mutated genes. Important deregulated genes are indicated in blue, red and green.

In addition, we analyzed NKL-code member MSX1 which was found to be overexpressed in subsets of HSTL patients and in HSTL-derived sister cell lines DERL-2 and DERL-7 which were chosen as models. Our experimental data are summarized in Figure 9, showing deduced regulatory gene networks located upstream of MSX1 imputed to both cell lines. Copy-number gains and subsequent overexpression of AUTS2 (located at 7q11) was correlated with the appearance of the HSTL cytogenetic hallmark rearrangement i(7q). Of note, two additional genes both potential targets of the aberrant i(7q) have been described recently [39]. AUTS2 is an activator of MSX1 and operates via PRC1.5 [15, 18]. Histone HIST1H3B was more intensively expressed and contained the mutation K27M which prevents suppressive tri-methylation performed by EZH2 [29]. Homeodomain protein PITX1 activated the transcription of MSX1 and might be regulated by SETD2 via STAT1. A loss-of-function mutation of SETD2 and its reduced expression level contributed to PITX1 activation. NKX2-2 operated directly as mutual activator. DERL-2 contains a partial chromosomal duplication of 14q which results in copy-number gains and subsequent overexpression of BMP4. BMP4 is an activator of the BMP-pathway which repressed MSX1 expression. PDGFRB operated as inhibitor of BMP4 in DERL-7 while the aberrant fusion gene CD53-PDGFRB activated BMP4 expression in DERL-2. Thus, both cell lines express the MSX1-activators AUTS2, mutated HIST1H3B, NKX2-2 and PITX1 but differ in their levels of MSX1-inhibitor BMP4.

Karyotyping and genome-sequencing identified a novel rearrangement in DERL-2, t(1;5)(p13;q32), which resulted in formation of the fusion gene CD53-PDGFRB. Several PDGFRB fusion genes have been detected in myeloid and rarely in lymphoid maligancies [28, 40, 41]. Reported fusion-partners include BCR, CEV14, ETV6 and HIP1 and act as receptor-activators via oligomerization and as expression-drivers of the fusion genes [28]. CD53 encodes a transmembrane protein of the tetraspanin-family and is expressed in multiple hematopoietic cell types including T-cells and NK-cells [42]. The expression level of CD53 was higher in normal NK-cells than in T-cells, showing NK-cell specific activity and explaining its function as a plausible expression-driver. The expression level of wild type PDGFRB in DERL-7 resembled that in normal NK-cells, indicating that this pathway also operates in this type of lymphocytes. Interestingly, aberrant autocrine activation of PDGFRB has been shown in large granular lymphocyte leukemia which presents as T-cell or NK-cell malignancy [43], highlighting the role of PDGFRB in the context of deregulated T-/NK-cell differentiation. Its inhibitory effect on BMP4 and subsequent activation of MSX1 suggests that PDGF-signalling contributes to MSX1 expression in normal NK-cell differentiation [19]. T-cell development is also influenced by BMP-signalling with BMP4 repressing early steps in differentiation [44]. Aberrant suppression of BMP-signalling mediates MSX1 activation and disturbs T-cell differentiation in T-ALL [12]. The MSX1-activator AUTS2 probably plays a regulatory role in normal NK-cells and shows aberrant activation in T-ALL as well [15]. Thus in summary, overexpression of AUTS2, rearrangement of CD53 and PDGFRB, and subsequent deregulation of MSX1 affect NK-cell specific genes in HSTL-derived cell lines DERL-2/7.

Post-translational modifications of histones are basic to gene regulation. In particular, tri-methylation of histone H3 at position K27 is performed by EZH2 and effects transcriptional repression. H3K27-mutations including H3(K27M) inhibit EZH2 activity and thereby decrease global levels of repressive H3K27me3 [29, 45]. Thus, HIST1H3B(K27M) generates aberrantly active chromatin. This type of mutation has been described in acute myeloid leukemia as well, showing its widespread involvement in hematopoietic malignancies [46]. Decreased expression levels or loss-of-function mutations of EZH2 have similar effects and play a role in T-cell leukemia [47, 48]. Consistently, mutated H3 or EZH2 perform aberrant H3K27me3 redistribution, inhibit cell differentiation and support the generation of cancer [49]. Interestingly, in glioma mutated H3(K27M) is also associated with the PDGF-pathway [50, 51]. Moreover, in this cancer MSX1 acts as a tumor suppressor, suggesting that the expression of MSX1 might be more generally regulated by this pathway [52]. MSX1 in turn recruits PRC2 and regulates target genes via H3K27me3 [53]. Therefore, histone H3-mutations may impact MSX1 regulated gene activities twofold.

Our results revealed the roles of homeobox genes NKX2-2 and PITX1 in HSTL which serve as oncogenes in T-ALL [7, 31, 54]. NKX2-2 belongs to the NKL homeobox gene subclass but is not a member of the hematopoietic NKL-code. In addition to other potential oncogenic activities of NKX2-2 we identified a directly activating impact on MSX1 transcription. Recently, ectopic expression of NKX2-2 has been described in Hodgkin lymphoma [55], indicating oncogenic functions in both B- and T-lymphocytes. However, aberrant NKX2-2 expression was not identified in the group of HSTL patients analyzed here, suggesting that this oncogene is rarely activated in this cancer type. PITX1 belongs to the PRD-subclass of homeobox genes. Our data indicated that PITX1 was regulated by SETD2 and STAT1 and contributed to activation of MSX1 expression. In T-ALL PITX1 activates MSX1 via BMP-pathway inhibitor CHRDL1 [12]. This relationship was not detected in HSTL-derived cell lines DERL-2/7 (not shown), implying activation of MSX1 expression in lymphoid malignancies by alternative mechanisms.

In this study, we also identified several additional cancer genes: STAT5B mutated at N642H which displays oncogenic aberrations in both T-cell lymphoma and T-ALL [27, 56, 57]; KDM7A a histone demethylase which may support aberrant activities of mutated histone H3 and of AUTS2 [58] while the effect of the mutation P897L remains unclear hitherto. TLE1 is a repressive cofactor which interacts with NKL homeodomain TFs via their NKL-specific en1-domain [59–61]. It would be interesting to see if the mutation P287A impacts this interaction and the subsequent target gene activities. SLFN13 is in addition to other SLFN family members a tRNAse which performs inhibition of proliferation and represents, therefore, a tumor suppressor gene [62]. Here, we identified a targeted deletion of SLFN13 in DERL-7, indicating that this gene basically impacts growth control in HSTL as well. Finally, detection of deleted tumor suppressor CDKN2A in both DERL-2 and DERL-7 further highlights the role of deregulated proliferation in this T-cell lymphoma entity.

HSTL-derived cell lines DERL-2/7 are malignant T-cells and express apposite markers [24]. Furthermore, we have shown these cell lines to bear several characteristics which are typical for this type of T-cell lymphoma including the rearranged chromosome i(7q) and specific mutations in HIST1H3B, KDM7A, SETD2 and STAT5B [26, 27]. However, in addition to the expression-based clustering results we identified several activated genes which are associated with NK-cell development including AUTS2, CD53, ETS1, ID2, MSX1, PDGFRB and TBX21 [3, 19]. Therefore, these data indicate that in cell lines DERL-2/7 and thus in HSTL patients, early differentiation processes separating T-cell and NK-cell lineages are disturbed and generate tumor cells which possess characteristics of both lineages. Moreover, our data indicated that NKL homeobox gene MSX1 shows dual oncogenic and tumor suppressor activities, reflecting T-ALL and NK-cell leukemia characteristics, respectively [12, 19]. Accordingly, the T-cell line LOUCY is derived from ETP-ALL which also expresses T-cell markers and NK-cell genes including AUTS2, ID2 and MSX1 [19, 47, 48]. Furthermore, malignant ALCL cells resemble normal ILC3 cells [63], displaying a developmental status located between T-cells and the closely related innate lymphocytes. The identification of such developmental hybrids might permit therapeutically exploitation by performing pharmacologically enforced terminal differentiation of the malignant cells.

Tumors are not constant. They evolve by the generation of genetic diversity and subsequent natural selection of subclones best able to resist host defence and treatment [64]. Therefore, the previously reported and here identified differences between sister cell lines DERL-2 and DERL-7 may reflect such evolutionarily formed genetic diversity. Mutations consisting in both cell lines were generated early in the course of tumorigenesis and include i(7q) targeting AUTS2, loss-of-function mutation of SETD2, deletion of both alleles of CDKN2A, and deletion of one allele of SLFN13. Mutations consisting in just one cell line represent late aberrations and include dup(14q) targeting BMP4, and t(1;5)(p13;q32) resulting in the CD53-PDGFRB fusion gene which increases BMP4 expression as well. Accordingly, early mutations activate MSX1 expression (T-cell oncogene) in addition to cell proliferation - late mutations mediate inhibition of MSX1 (NK-cell tumor suppressor). Therefore, these data indicate dedifferentiation from the T-cell status and an increase of NK-cell characteristics in the course of HSTL development.

The phenomenon of tumor evolution has been described in other cell lines as well including Hodgkin lymphoma sister cell lines HDLM-1 and HDLM-2, diffuse large B-cell lymphoma subclones U-2932-R1 and U-2932-R2, and cutaneous T-cell lymphoma sister cell lines MAC-1, MAC-2A, MAC-2B [65–67]. These models in addition to DERL-2 and DERL-7 may help to investigate the molecular processes generating genetic differences in tumorigenesis and to test novel therapeutic treatments.

Taken together, our data revealed deregulated NKL homeobox gene MSX1 in HSTL. By analyzing aberrant mechanisms involved in MSX1 expression we recognized differences in the sister cell lines DERL-2 and DERL-7 which might represent consequences of clonal selection or intratumoral diversity. In the evolutionary course of this tumor the malignant cells shifted their identity from the T-cell status back to progenitor cells, gaining NK-cell characteristics. These results may support the understanding of the biology of HSTL tumors and may assist to develop novel therapeutic options in the future.

## MATERIALS AND METHODS

### Expression profiling

Public expression profiling datasets used in this study were generated by U133 Plus 2.0 gene chips from Affymetrix and obtained from Gene Expression Omnibus (GEO, https://www.ncbi.nlm.nih.gov). We exploited datasets GSE6338, GSE19069, GSE57944 and GSE19067 for examinations of T-cell lymphoma entities. In datasets GSE6338 and GSE19069 outliers and/or statistically significantly higher values of the median was defined as overexpression. In datasets GSE57944 and GSE19067 we set a cut-off at 3.0 and 8.0, respectively, to identify patient samples showing overexpression.

For clustering of cell lines according to gene expression profiling data we used datasets generated by Prof. Andreas Rosenwald (Institute of Pathology, University of Würzburg, Germany), by Dr. Robert Geffers (Genome Analytics, Helmholtz Centre for Infection Research, Braunschweig, Germany), and retrieved from GEO datasets GSE128302 (T-cell lymphomas), GSE87334 (T-ALLs), GSE19067 (IMC-1, KHYG1, SNK6) and GSE53478 (NK-92). After RMA-background correction and quantile normalization of the spot intensities, gene expression values were subsequently log2 transformed. Data processing was performed via R/Bioconductor (version 3.3.2/3.3, https://www.bioconductor.org) using limma and affy packages. The generation of the dendrogram was performed via hierarchical clustering by the Ward‘s method which was applied on the Euclidean distance matrix.

For creation of heat maps showing selected values of gene expression profiling data we used the software CLUSTER (version 2.11) and TREEVIEW (version 1.60) originally developed by Michael Eisen (http://bonsai.hgc.jp/~mdehoon/software/cluster/index.html).

### Cell lines and treatments

The cell lines DERL-2, DERL-7, KHYG-1, NK-92, YT, and LOUCY were obtained from the DSMZ (Braunschweig, Germany) and cultivated as described elsewhere [24, 68]. The NK-cell line IMC-1 was kindly obtained from Dr. I. Ming Chen (Albuquerque, NM), NKL from Dr. Jerome Ritz (Boston, MA), and SNK-6 from Dr. N. Shimizu (Tokyo, Japan) [69, 70, 71]. To modify gene expression levels we used gene specific siRNA oligonucleotides in comparison to AllStars negative Control siRNA (siCTR) which were obtained from Qiagen (Hilden, Germany). SiRNAs (80 pmol) were transfected into 1x10^6^ cells by electroporation using the EPI-2500 impulse generator (Fischer, Heidelberg, Germany) at 350 V for 10 ms. Electroporated cells were harvested after 20 h cultivation. Cells were variously treated for 16 h with 20 ng/ml recombinant BMP4, with 1 or 10 ng/ml recombinant PDGFD (R & D Systems, Abingdon, UK), with 100 μM Dasatinib (LC Laboratories, Woburn, MA), with 5 μM BMP receptor inhibitor dorsomorphin (DM) (Calbiochem, Darmstadt, Germany) dissolved in dimethyl sulfoxide (DMSO), with 10 μM EZH2-inhibitor DZNep (Sigma, Taufkirchen, Germany), or with 10 μg/ml histone deacetylase inhibitor trichostatin A (TSA) (Sigma).

### Polymerase chain-reaction (PCR) analyses

Total RNA was extracted from cultivated cell lines using TRIzol reagent (Invitrogen, Darmstadt, Germany). Primary human total RNA was commercially obtained. We used RNA from T-cells and NK-cells obtained from Biochain/BioCat (Heidelberg, Germany), and RNA from HSCs obtained from Miltenyi Biotec (Bergisch Gladbach, Germany). cDNA was synthesized using 5 μg RNA, random priming and Superscript II (Invitrogen). Real-time quantitative (RQ)-PCR analysis was performed using the 7500 Real-time System and commercial buffer and primer sets (Thermo Fisher, Darmstadt, Germany). Quantification of MSX1 was performed as described recently [12]. For normalization of expression levels we quantified the transcripts of TATA box binding protein (TBP). Quantitative analyses were performed twice in triplicate. Standard deviations are presented in the figures as error bars. Statistical significance was assessed by Student´s *T*-Test and the calculated *p*-values were indicated by asterisks (^*^p < 0.05, ^**^p < 0.01, ^***^p < 0.001, n.s. not significant).

Reverse-transcription (RT)-PCR analysis was performed using Taq-DNA polymerase (Qiagen) and thermocycler TGradient (Biometra, Göttingen, Germany). The oligonucleotides were obtained from Eurofins MWG (Ebersberg, Germany) and their sequences were as follows: CD53-for 5′-TCTGTGTTACCAGCCTTGTCTCG-3′, CD53-rev 5′-GACAAACACATTGCCCAGCGTG-3′, PDGFRB-for 5′-ACACTGCGTCTGCAGCACGTGG-3′, PDGFRB-rev 5′-GGAGTCATAGGGCAGCTGCATG-3′. The generated PCR products were analyzed by agarose gel electrophoresis using Gene Ruler 100 bp Plus (Thermo Fisher) as marker.

### Protein analyses

Western blots were generated by the semi-dry method. Protein lysates from cell lines were prepared using SIGMAFast protease inhibitor cocktail (Sigma). Proteins were transferred onto nitrocellulose membranes (Bio-Rad, München, Germany) and blocked with 5% dry milk powder dissolved in phosphate-buffered-saline buffer (PBS). The following antibodies were used: MSX1 (R & D Systems), alpha-Tubulin (Sigma), PDGFRB (R & D Systems), phospho-PDGFRB (Aviva Systems Biology, Eching, Germany), NKX2-2 (Aviva Systems Biology) and PITX1 (Abnova, Taipei, Taiwan). For loading control blots were reversibly stained with Poinceau (Sigma) and detection of alpha-Tubulin (TUBA) was performed thereafter. Secondary antibodies were linked to peroxidase for detection by Western-Lightning-ECL (Perkin Elmer, Waltham, MA, USA). Documentation was performed using the digital system ChemoStar Imager (INTAS, Göttingen, Germany). PDGFD and BMP4 were quantified in the medium by ELISA using according Quantikine ELISA kits from R & D Systems. Samples were obtained by harvesting supernatants of 1x10^6^ cells which were washed in PBS and subsequently incubated in 1 ml medium for 24 h.

### Chromosomal and genomic analyses

The karyotypes of DERL-2 and DERL-7 were generated as described previously [72]. For genomic profiling and sequencing the genomic DNA of cell lines was prepared by the Qiagen Gentra Puregene Kit (Qiagen). Labelling, hybridization and scanning of Cytoscan HD arrays was performed at the Genome Analytics Facility, Helmholtz Centre for Infection Research, according to the manufacturer´s protocols (Affymetrix, High Wycombe, UK). Data were interpreted using the Chromosome Analysis Suite software version 3.1.0.15 (Affymetrix).

Genomic sequencing was performed as follows: Standard genomic library preparation and sequencing were conducted at GATC Biotech (Konstanz, Germany). The libraries were sequenced on Illumina HiSeq2500 (2 × 151 cycles, paired end run) with >300 million reads per sample for a coverage of 30-fold. Reads were quality controlled via FastQC (version 0.11.5, https://www.bioinformatics.babraham.ac.uk/projects/fastqc) and trimmed via fastq-mcf (ea-utils 1.04.807). The data have been deposited in the ArrayExpress database at EMBL-EBI (https://www.ebi.ac.uk/arrayexpress) via accession number E-MTAB-7734. For detection of gene mutations the reads were aligned by STAR (version 2.5.3a) to the Gencode Homo sapiens genome (version 26) and converted/sorted via samtools (version 0.1.19) [73, 74]. Duplicates were removed (picard version 2.9.2), and variants called via GATK tools (version 3.7) and overlapping VarScan (version 2.4.3) results [75, 76]. Mutation effects were annotated via the Ensembl VEP (release-89, GRCh38) [77]. Data were processed and analyzed in the R/Bioconductor environment (version 3.3.2/3.3, https://www.bioconductor.org). Genomic structural variants were detected via seeksv (version 2.0) and lumpy [78, 79].

### Sanger sequencing

For confirmation of identified mutations we performed Sanger sequencing of cDNA samples. DNA-fragments were generated by PCR using the following oligonucleotides: HIST1H3B-for 5′-ATGGCTCGTACTAAACAGACAGC-3′, HIST1H3B-rev 5′-AGAGCCTTTGGGTTTTAAGACTG-3′, KDM7A-for 5′-GTAGGAATTATGTGGACAGCAG-3′, KDM7A-rev 5′-TATACACACAAACTGCTCCAGG-3′, SETD2-for 5′-CATGGACAGTGCAATCTCTGATG-3′, SETD2-rev 5′-AACTGTCCAGGAGTTTGGTGGC-3′, STAT5B-for 5′-AGGACGGAATTACACTTTCTGG-3′, STAT5B-rev 5′-ATCTGTGGCTTCACGTATCCATC-3′, TLE1-for 5′-GTGATGGTGACAAAAGCGATGAC-3′, TLE1-rev 5′-CAAAAGGAGCAGGATATGGGCC-3′. PCR products were treated using exonuclease 1 and alkaline phosphatase Illustra ExoProStar according to the recommended protocol for a 5 μl aliquot (GE Healthcare Life Sciences, Freiburg, Germany). The sequencing reactions were performed using BigDye Terminator v3.1 Cycle Sequencing Kit (Thermo Fisher) for 25 cycles in a Veriti Thermal Cycler (Thermo Fisher). For purification we used CleanSEQ reagent in combination with the Agencourt CleanSEQ magnetic plate (Beckman Coulter, Krefeld, Germany). The beads were eluted with 40 μl HiDi formamide and applied to the ABI 24 capillary 3500XL automated DNA sequencer (Thermo Fisher).

### Reporter gene assay

For creation of a reporter gene construct we combined a reporter with a regulatory genomic fragment derived from the upstream region of MSX1, containing a consensus binding site for NKX2-2 [80]. We cloned the genomic PCR product of the corresponding upstream region (regulator) and of the HOXA9 gene, comprising exon1-intron1-exon2 (reporter), into the *Hind*III/*Bam*HI and *Eco*RI sites, respectively, of the expression vector pcDN A3 downstream of the CMV enhancer. The oligonucleotides used for the amplification of the NKX2-2 site were obtained from Eurofins MWG (Ebersbach, Germany). Their sequences were as follows: MSX1-for 5′-CC AAGCTTCAGGCAGATCTTGCATCTCC-3′, MSX1-rev 5′-ATGGATCCTTATCCTAGGAGAAAGACATACTAT TAAC-3′, HOXA9-for 5′-TGGCATTAAACCTGAA CCGC-3′, HOXA9-rev 5′-ACTCTTTCTCCAGTTC CAGG-3′. Introduced restriction sites used for cloning are underlined. Transfections of gene expression construct for NKX2-2 (cloned in vector pCMV6-XL4 and obtained from Origene, Wiesbaden, Germany) into NIH-3T3 cells was performed using SuperFect Transfection Reagent (Qiagen). Commercial HOXA9 and TBP assays (Thermo Fisher) were used for RQ-PCR to quantify the spliced reporter-transcript, corresponding to the regulator activity.

## SUPPLEMENTARY MATERIALS






