# Supplementary material for: Deregulated expression of NKL homeobox genes in T-cell lymphomas
Source: Oncotarget. 2019 May 14;10(35):3227–47. doi: 10.18632/oncotarget.26929 (PMC6524933; doi:10.18632/oncotarget.26929)
Supplement: Supplementary file 1 [file oncotarget-10-3227-s001.pdf]

Deregulated expression of NKL homeobox genes in T-cell lymphomas

SUPPLEMENTARY MATERIALS

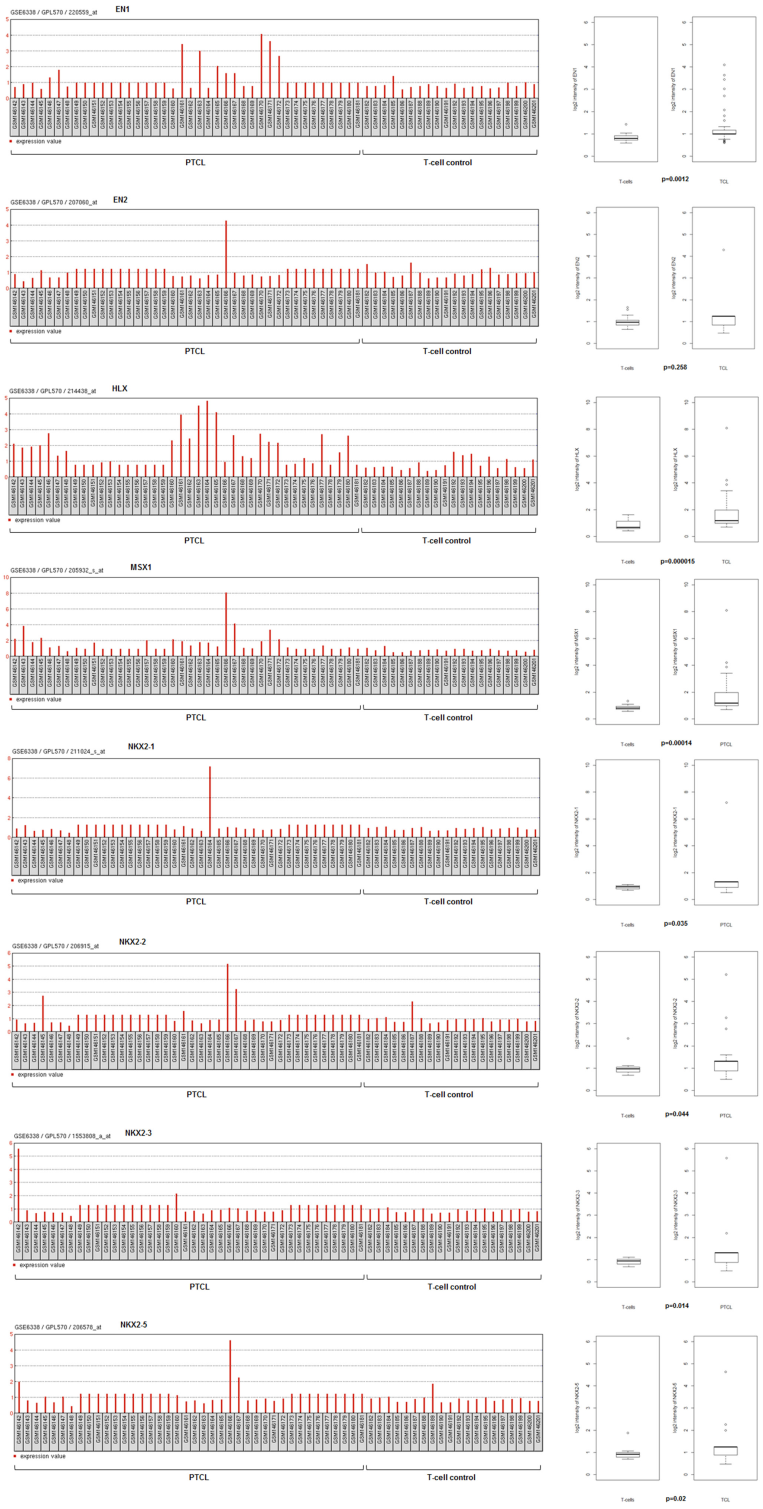

Supplementary Figure 1: Screening of PTCL patients in comparison to T-cell controls using dataset GSE6338. Boxplots and calculated *p*-values indicate samples showing particular NKL homeobox gene overexpression.

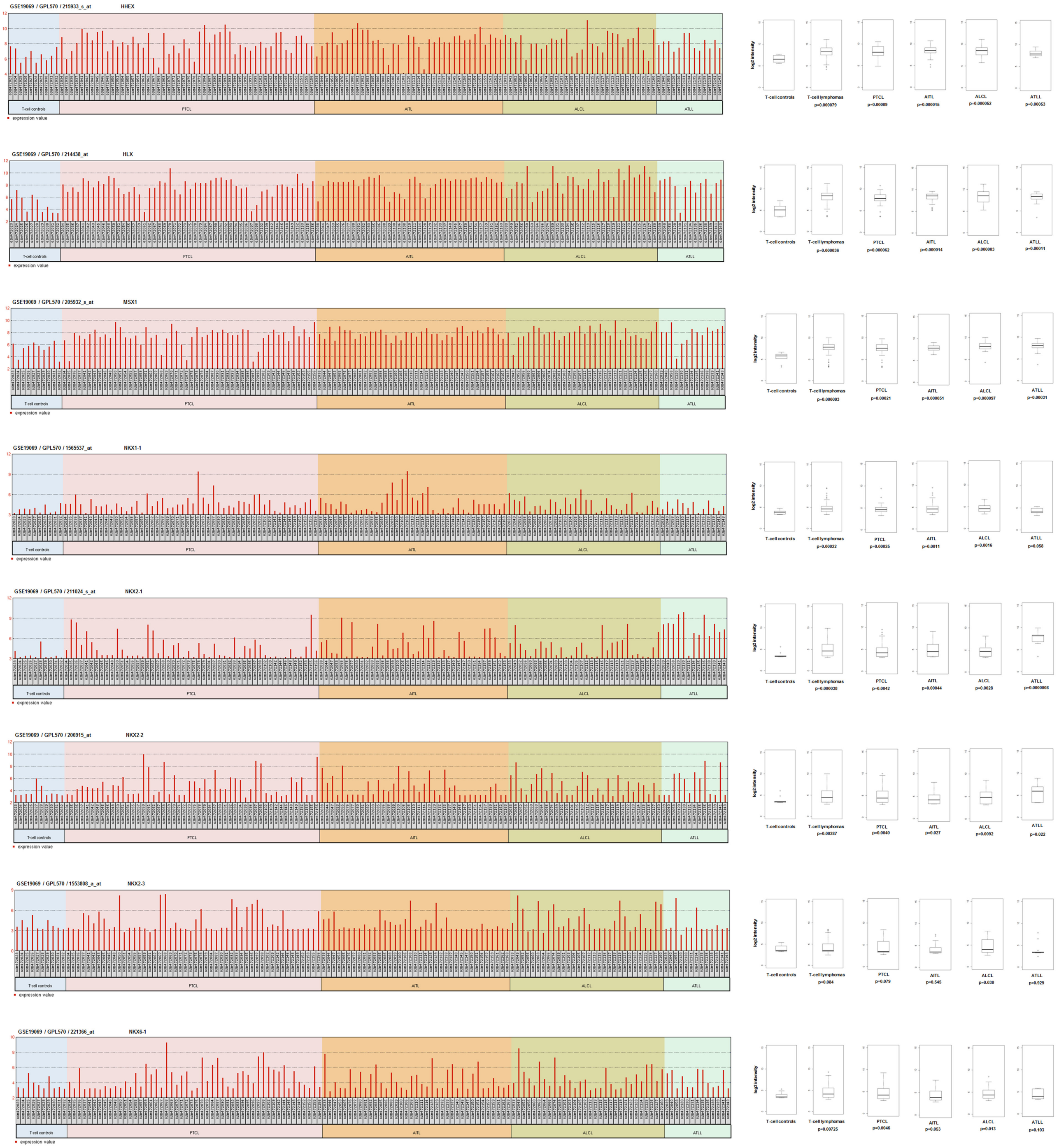

Supplementary Figure 2: Screening of PTCL, AITL, ALCL, and ATLL patients in comparison to T-cell controls using dataset GSE19069. Boxplots and calculated *p*-values indicate samples showing particular NKL homeobox gene overexpression.

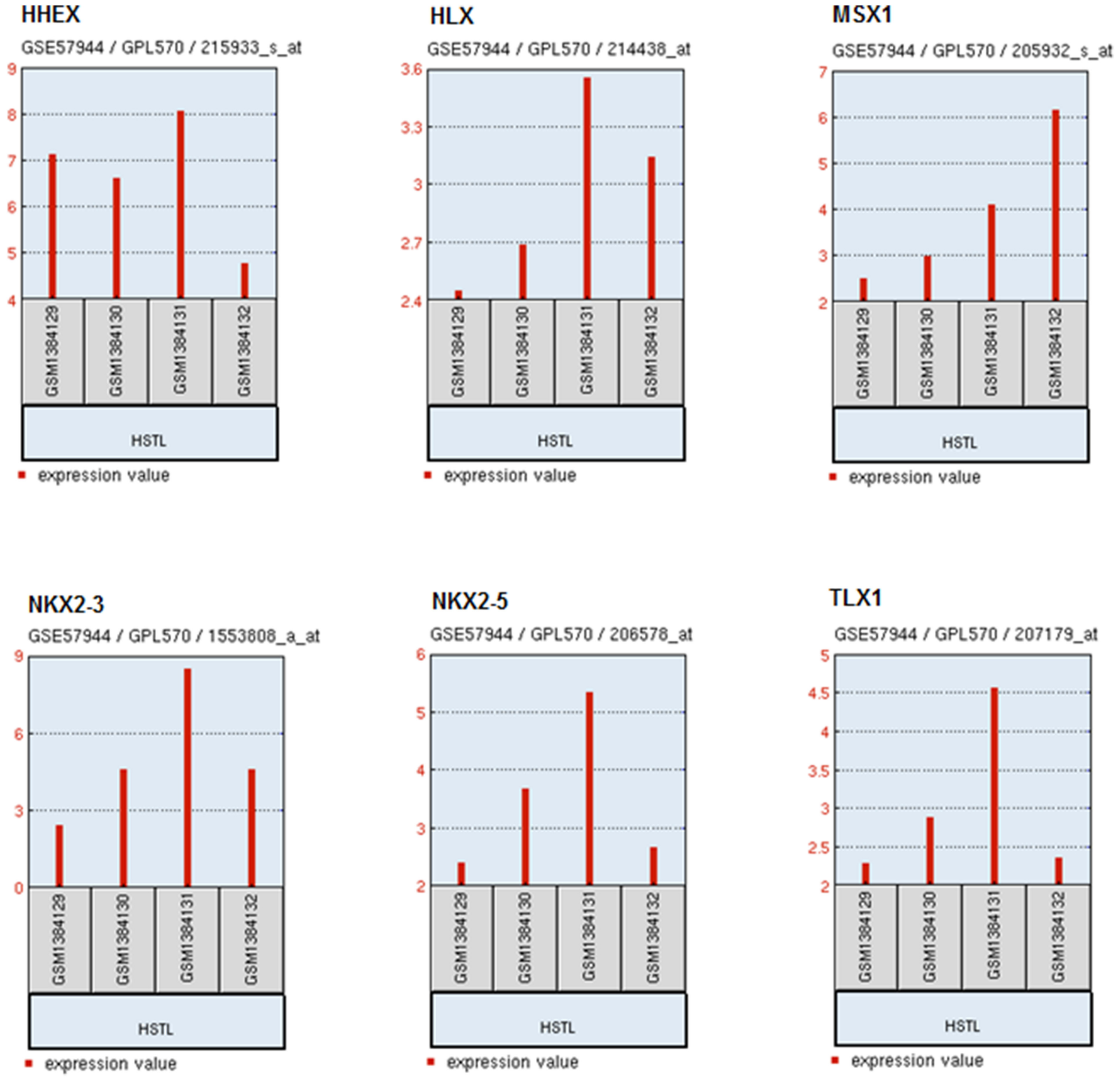

Supplementary Figure 3: Screening of HSTL patients using dataset GSE57944. We have set a cut-off at 3.0 to identify overexpressed NKL homeobox genes.

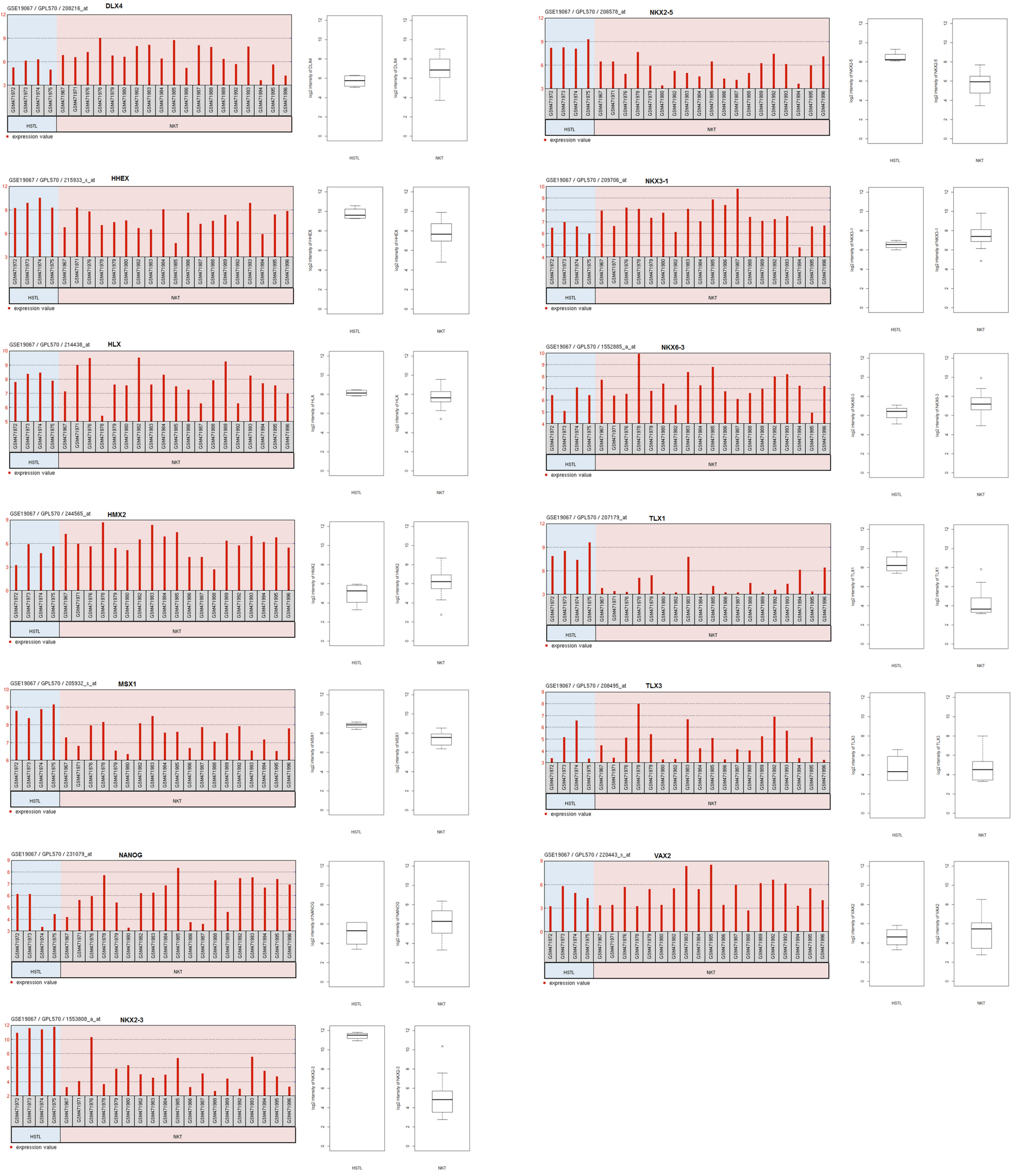

Supplementary Figure 4: Screening of HSTL and NKTL patients using dataset GSE19067. We have set a cut-off at 8.0 to identify overexpressed NKL homeobox genes.

Hematopoietic cell lines (GSE57083)

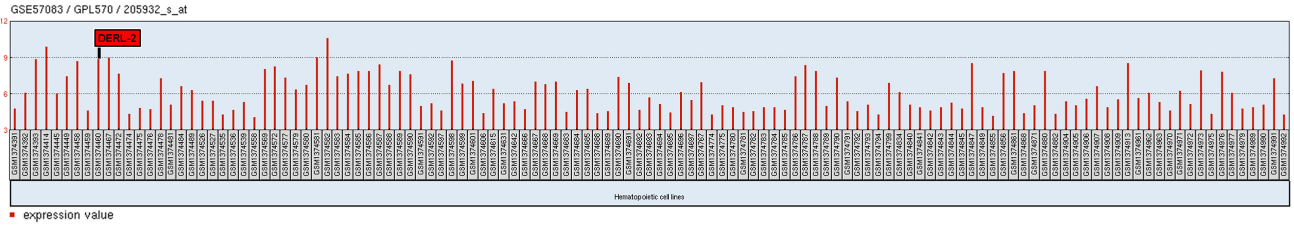

GSM1374391	AML143D10	4.81264
GSM1374392	AMO-1	6.11963
GSM1374393	ARH-77	8.87046
GSM1374414	BV-173	9.92991
GSM1374445	CCRFCEM	6.04892
GSM1374449	CMK	7.45867
GSM1374458	CTV-1	8.72803
GSM1374459	DAUDI	4.67645
GSM1374460	DERL-2	8.8694
GSM1374467	DOHH-2	8.99924
GSM1374472	EHEB	7.70832
GSM1374474	EM-2	4.40102
GSM1374475	EOL-1	4.84993
GSM1374476	ESKOL	4.77991
GSM1374478	F36P	7.33344
GSM1374481	FARAGE	5.13075
GSM1374484	GRANTA-519	6.66211
GSM1374489	HBL-1	6.33476
GSM1374526	HEL	5.49229
GSM1374527	HEL	5.4774
GSM1374535	HL-60	4.33215
GSM1374536	HL-60	4.69374
GSM1374539	HLY-1	5.36601
GSM1374558	HT	4.13094
GSM1374569	HUT-78	8.08409
GSM1374572	IM9	8.29258
GSM1374577	JEKO	7.35558
GSM1374579	JJN3	6.40014
GSM1374580	JURKAT	6.77721
GSM1374581	JVM-2	9.01873
GSM1374582	JVM-3	10.6147
GSM1374583	K-562	7.45821
GSM1374584	KARPAS-1106P	7.69513
GSM1374585	KARPAS-1106P	7.88509
GSM1374586	KARPAS-231	7.93179
GSM1374587	KARPAS-231	8.43804
GSM1374588	KARPAS-422	6.77434
GSM1374589	KARPAS-620	7.92691
GSM1374590	KASUMI-1	7.61184
GSM1374591	KASUMI-3	5.00796
GSM1374592	KASUMI-6	5.23447
GSM1374597	KG-1	4.62743
GSM1374598	KG-1a	8.77724
GSM1374599	KIT-225	6.89455
GSM1374601	KMS12BM	7.10693
GSM1374606	KU-812	4.4223
GSM1374615	L-363	6.42297
GSM1374631	LP1	5.22318
GSM1374642	MC116	5.40607
GSM1374666	ME-1	4.75839
GSM1374667	MEC1	7.04611
GSM1374668	MEC2	6.83731
GSM1374669	MEG01	7.01275
GSM1374683	ML-2	4.55743
GSM1374684	MM1R	6.35095
GSM1374685	MM1S_	6.44897
GSM1374688	MOLM-13	4.41052
GSM1374689	MOLM-13	4.58289
GSM1374690	MOLM-16	7.39349
GSM1374691	MOLP-8	6.91705
GSM1374692	MONO-MAC-6	4.70305
GSM1374693	MUTZ-3	5.73012
GSM1374694	MV4-11	5.18767
GSM1374695	NAMALWA	4.46465
GSM1374696	NB-4	6.17113
GSM1374697	NB-4	5.53249
GSM1374767	NCLH929	7.00718
GSM1374774	NOMO-1	4.30754
GSM1374775	NUDHL-1	5.08407
GSM1374780	OCI-AML2	4.90301
GSM1374781	OCI-AML5	4.56474
GSM1374782	OCI-AML5	4.57837
GSM1374783	OCI-LY1	4.9491
GSM1374784	OCI-LY10	4.9149
GSM1374785	OCI-LY18	4.68498
GSM1374786	OCI-LY19	7.47108
GSM1374787	OCI-LY3	8.40235
GSM1374788	OCI-LY4	7.88233
GSM1374789	OCI-LY7	5.02041
GSM1374790	OCI-LY8	7.35858
GSM1374791	OCI-M1	5.41302
GSM1374792	OCI-M2	4.59335
GSM1374793	OCI-MY1	5.16752
GSM1374794	OCI-MY5	4.33919
GSM1374799	OPM-2	6.91938
GSM1374834	PL-21	6.1686
GSM1374840	RAJI	5.14113
GSM1374841	RAJI	4.90715
GSM1374842	RAJI	4.66174
GSM1374843	RAMOS	4.90284
GSM1374844	RAMOS	5.30295
GSM1374845	RC-K8	4.80161
GSM1374847	REH	8.55285
GSM1374849	RI-1	4.949
GSM1374855	RPMI-8226	4.22876
GSM1374856	RS4-11	7.76538
GSM1374861	SC-1	7.88348
GSM1374868	SET-2	4.43701
GSM1374871	SIGM-5	5.07958
GSM1374880	SKM-1	7.88591
GSM1374882	SKMM-2	4.40644
GSM1374904	SU-DHL-10	5.38712
GSM1374905	SU-DHL-2	5.08021
GSM1374906	SU-DHL-4	5.64823
GSM1374907	SU-DHL-5	6.68343
GSM1374908	SU-DHL-6	4.90413
GSM1374909	SU-DHL-8	5.56335
GSM1374913	SUP-B15	8.5666
GSM1374961	TF-1	5.69625
GSM1374962	THP-1	6.13944
GSM1374963	TMD-8	5.36639
GSM1374970	U-266	4.6452
GSM1374971	U-2932	6.26267
GSM1374972	U-2940	5.21515
GSM1374973	U-698-M	7.96703
GSM1374975	U-937	4.35866
GSM1374976	UKE-1	7.83757
GSM1374977	ULA	6.13273
GSM1374979	UT-7	4.82523
GSM1374989	WILL-1	4.92335
GSM1374990	WILL-2	5.11594
GSM1374991	WSU-DCL2	7.3259
GSM1374992	WSU-NHL	4.32096

Supplementary Figure 5: Screening of 123 hematopoietic cell lines for MSX1 overexpression using dataset GSE57083. Indicated is HSTL-derived cell line DERL-2 in the bar-plot diagram (above) and in the table listing the expression values (below).

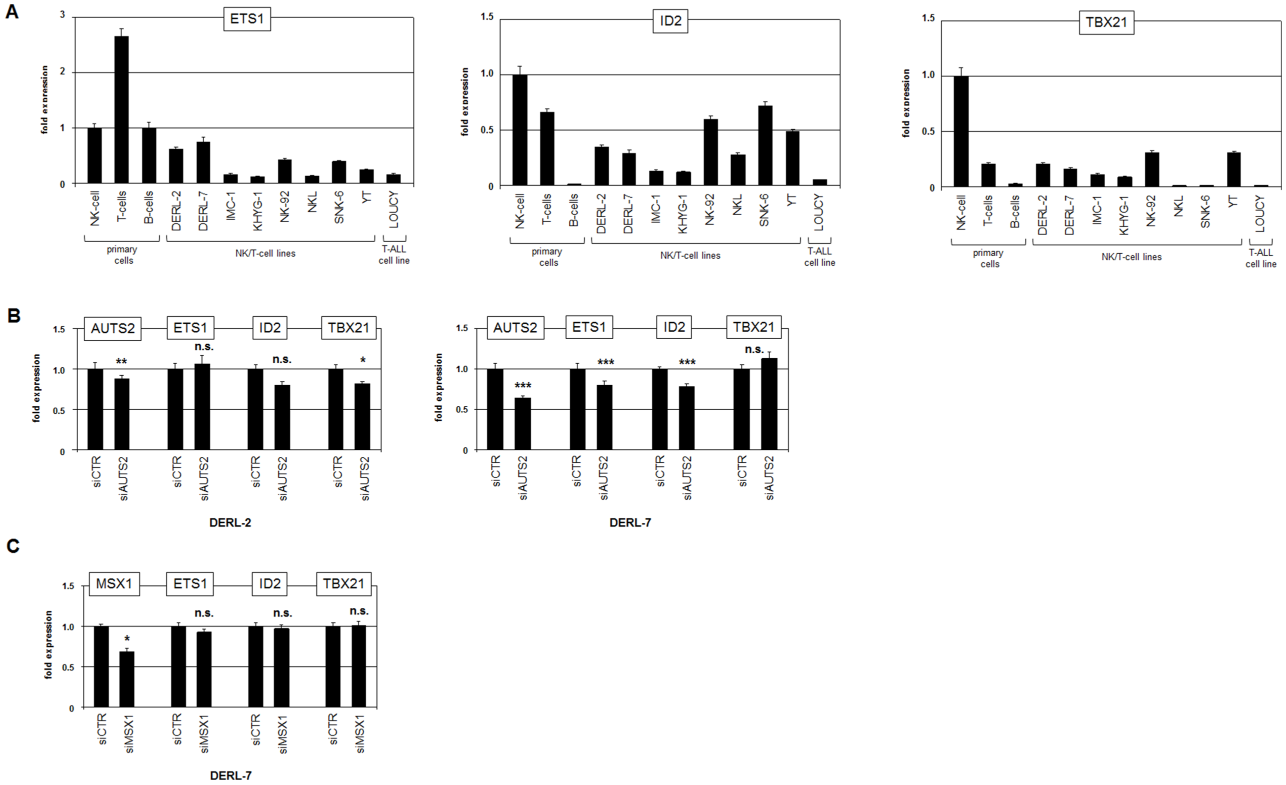

Supplementary Figure 6: (**A**) RQ-PCR analysis of ETS1, ID2, and TBX21 in primary cells and selected cell lines. RQ-PCR analysis of ETS1, ID2, and TBX21 in DERL-2/7 cells after treatment for siRNA-mediated knockdown of AUTS2 (**B**) and MSX1 (**C**).

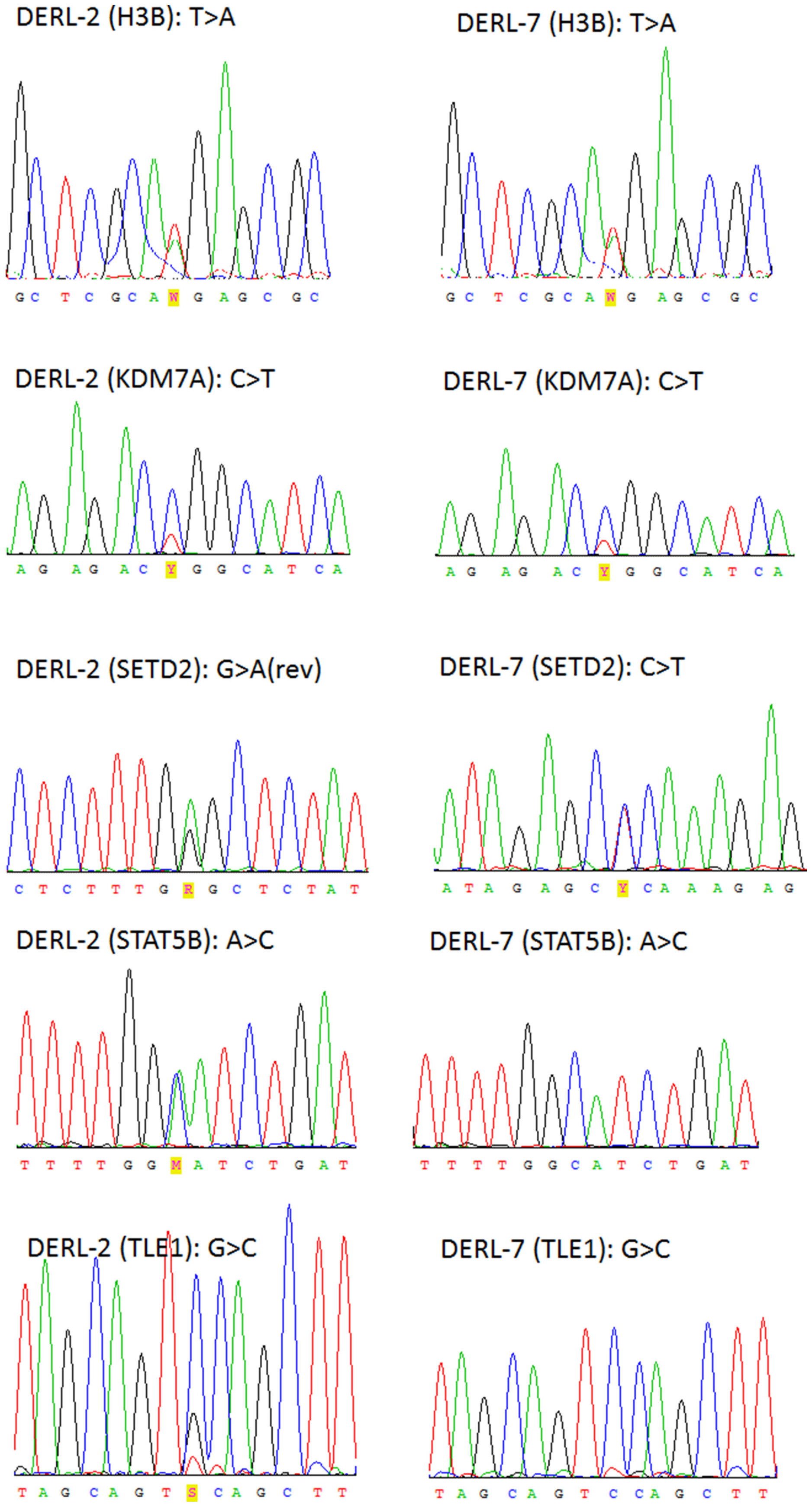

Supplementary Figure 7: Sanger sequencing data for HIST1H3B, KDM7A, SETD2, STAT5B, and TLE1 in DERL-2 and DERL-7.

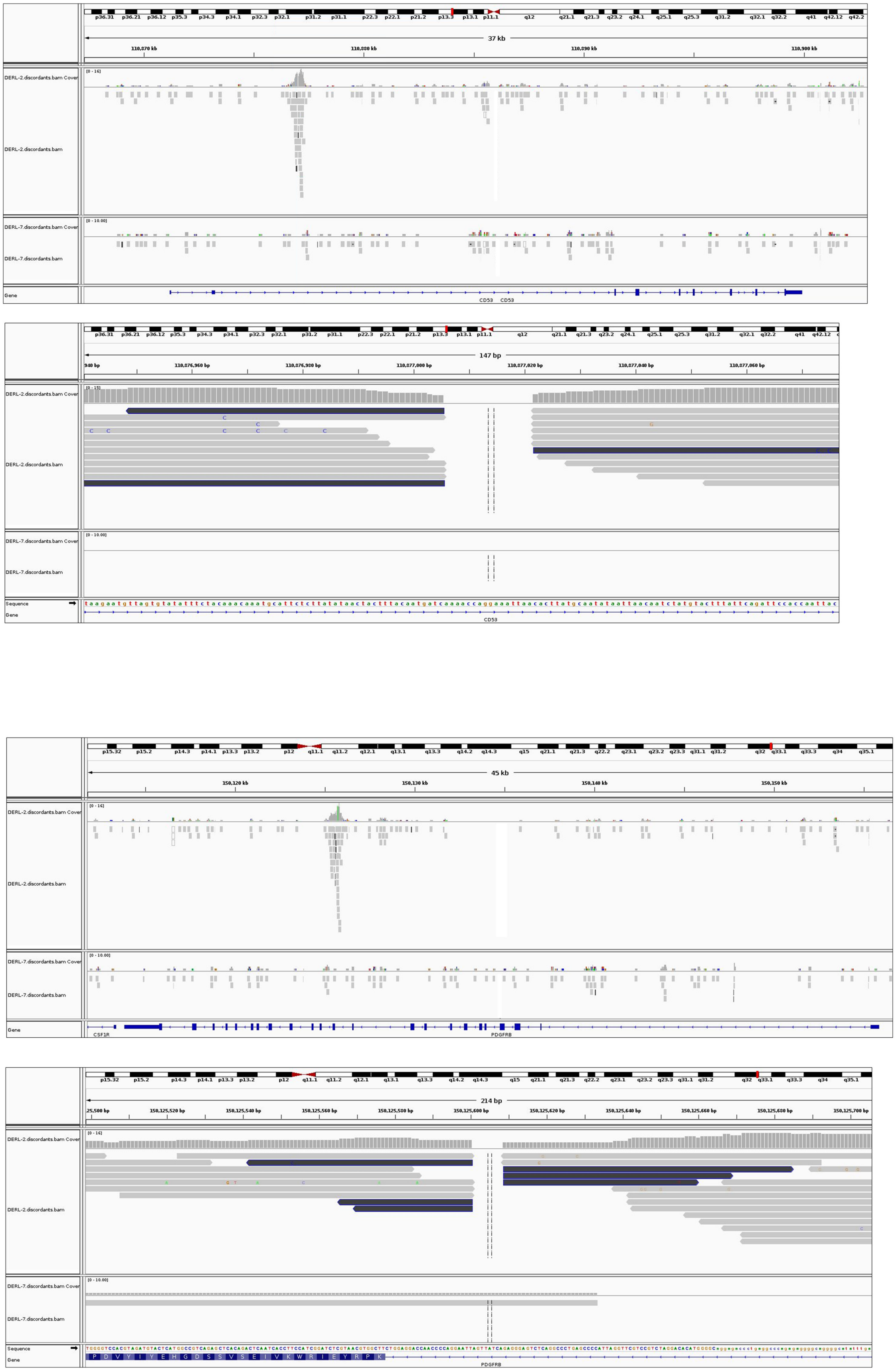

Supplementary Figure 8: Genome sequencing data for CD53 (above) and PDGFRB (below) of DERL-2 and DERL-7 indicating fusion gene CD53-PDGFRB in DERL-2.

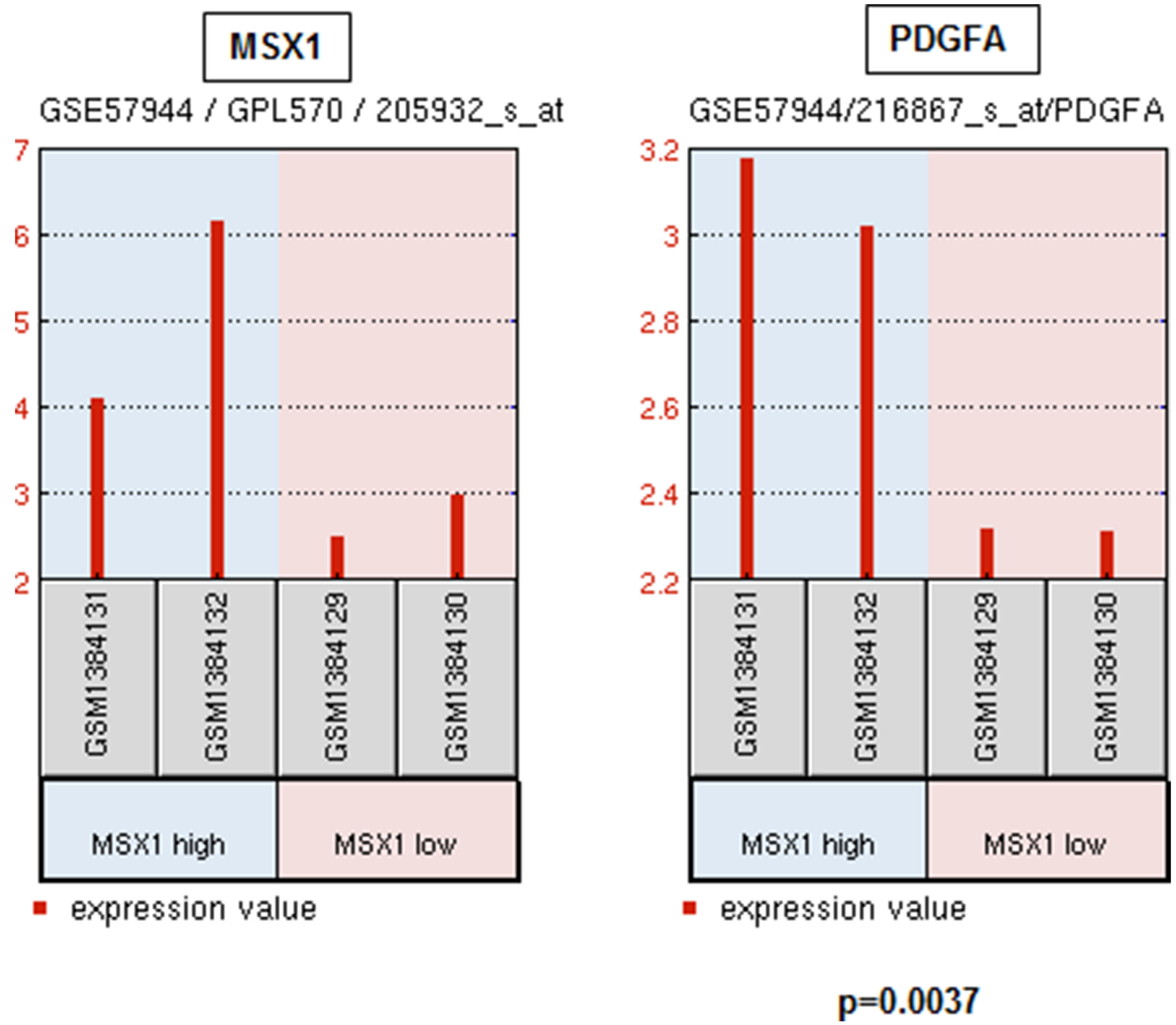

Supplementary Figure 9: Comparative expression profiling data of HSTL patients expressing MSX1-high and MSX1-low for MSX1 (left) and PDGFA (right). The *p*-value is indicated.

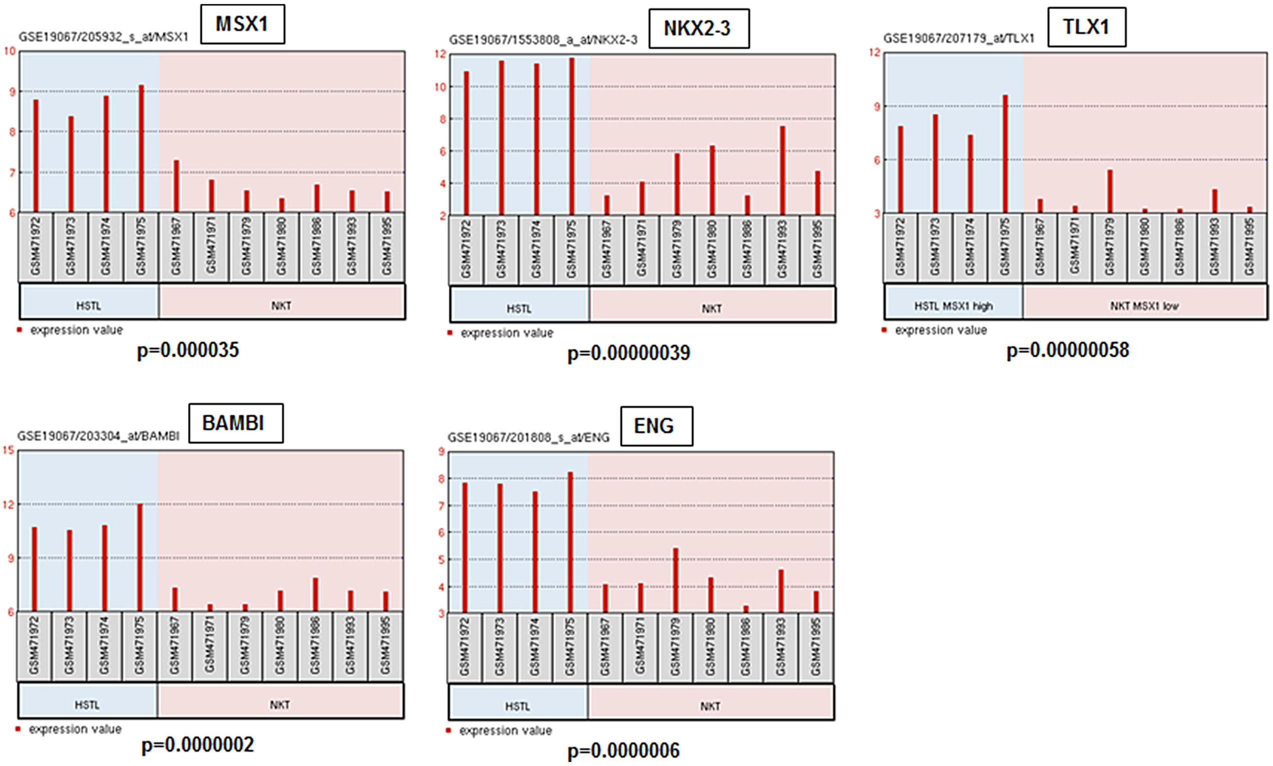

Supplementary Figure 10: Comparative expression profiling data HSTL- and NKTL-patients showing activities of MSX1, NKX2-3, TLX1, BAMBI and ENG. The *p*-values are indicated.

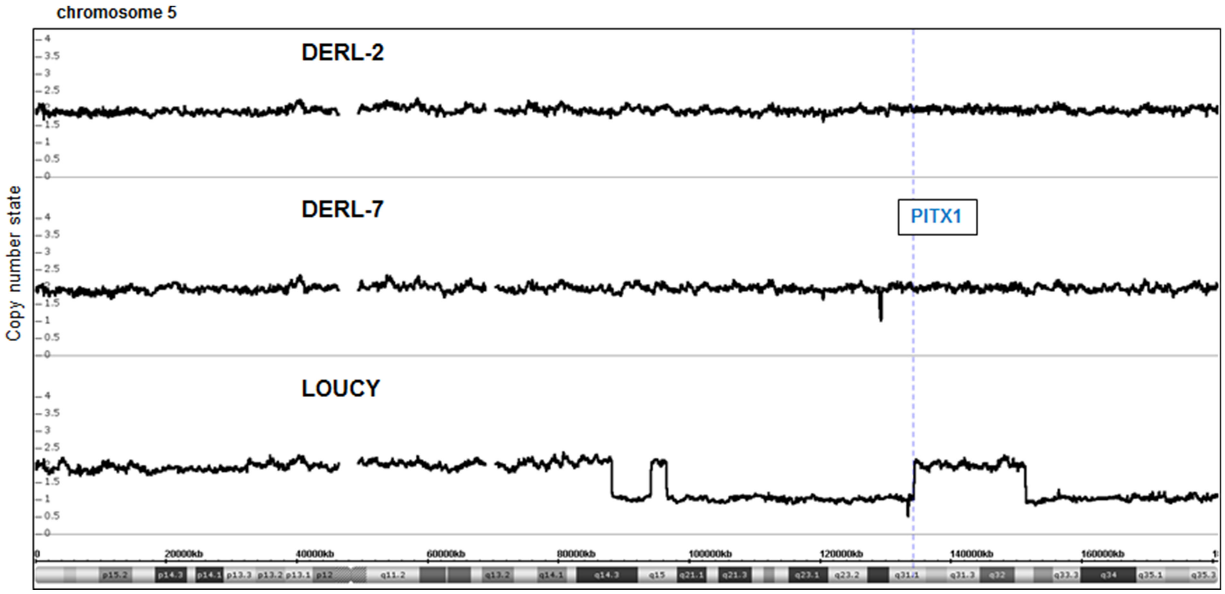

Supplementary Figure 11: Genomic profiling data of DERL-2, DERL-7 and LOUCY for chromosome 5, demonstrating a PITX1 downstream deletion just in LOUCY.

Supplementary Table 1: Comparative expression profiling data of DERL-2 and DERL-7. See Supplementary Table_1

Supplementary Table 2: Comparative expression profiling data of DERL-7 and selected NK-cell lines showing the overexpressed genes in DERL-7. See Supplementary Table_2
